# Disruption of KLHL6 Fuels Oncogenic Antigen Receptor Signaling in B-Cell Lymphoma

**DOI:** 10.1158/2643-3230.BCD-23-0182

**Published:** 2024-04-17

**Authors:** Leo Meriranta, Selma Sorri, Kanutte Huse, Xiaonan Liu, Ivana Spasevska, Sadia Zafar, Iftekhar Chowdhury, Olli Dufva, Eerika Sahlberg, Luka Tandarić, Marja-Liisa Karjalainen-Lindsberg, Marko Hyytiäinen, Markku Varjosalo, June H. Myklebust, Sirpa Leppä

**Affiliations:** 1 Research Programs Unit, Applied Tumor Genomics, Faculty of Medicine, University of Helsinki, Helsinki, Finland.; 2 Department of Oncology, Helsinki University Hospital Comprehensive Cancer Center, Helsinki, Finland.; 3 iCAN Digital Precision Cancer Medicine Flagship, Helsinki, Finland.; 4 Department of Cancer Immunology, Institute for Cancer Research, Oslo University Hospital, Oslo, Norway.; 5 KG Jebsen Centre for B-cell malignancies and Precision Immunotherapy Alliance, Institute of Clinical Medicine, University of Oslo, Oslo, Norway.; 6 Institute of Biotechnology, HiLIFE Helsinki Institute of Life Science, University of Helsinki, Helsinki, Finland.; 7 Hematology Research Unit Helsinki, Helsinki University Hospital Comprehensive Cancer Center, Helsinki, Finland.; 8 Department of Pathology, Helsinki University Hospital, Helsinki, Finland.

## Abstract

Pathomechanisms that activate oncogenic B-cell receptor (BCR) signaling in diffuse large B-cell lymphoma (DLBCL) are largely unknown. Kelch-like family member 6 (*KLHL6*) encoding a substrate-adapter for Cullin-3-RING E3 ubiquitin ligase with poorly established targets is recurrently mutated in DLBCL. By applying high-throughput protein interactome screens and functional characterization, we discovered that KLHL6 regulates BCR by targeting its signaling subunits CD79A and CD79B. Loss of physiologic KLHL6 expression pattern was frequent among the MCD/C5-like activated B-cell DLBCLs and was associated with higher CD79B levels and dismal outcome. Mutations in the bric-a-brac tramtrack broad domain of KLHL6 disrupted its localization and heterodimerization and increased surface BCR levels and signaling, whereas Kelch domain mutants had the opposite effect. Malfunctions of KLHL6 mutants extended beyond proximal BCR signaling with distinct phenotypes from *KLHL6* silencing. Collectively, our findings uncover how recurrent mutations in *KLHL6* alter BCR signaling and induce actionable phenotypic characteristics in DLBCL.

**Significance:** Oncogenic BCR signaling sustains DLBCL cells. We discovered that Cullin-3-RING E3 ubiquitin ligase substrate-adapter KLHL6 targets BCR heterodimer (CD79A/CD79B) for ubiquitin-mediated degradation. Recurrent somatic mutations in the *KLHL6* gene cause corrupt BCR signaling by disrupting surface BCR homeostasis. Loss of KLHL6 expression and mutant-induced phenotypes associate with targetable disease characteristics in B-cell lymphoma.

*See related commentary by Leveille et al.*

*See related commentary by Corcoran et al.*

## Teaser

Proteomic screening and functional investigation of E3 ubiquitin ligase substrate-adapter *KLHL6* reveal a new role targeting B-cell receptor, which is disrupted in a subset of B-cell lymphomas with targetable phenotypic characteristics.

## Introduction

Diffuse large B-cell lymphoma (DLBCL) is the most common hematologic malignancy in the western world. Although anthracycline-based chemoimmunotherapy is curative in the majority of patients, about one-third of the patients experience relapse with dismal outcome (1). Despite the discovery of multiple compounds that are lethal to a subset of B-cell lymphomas, successful precision medicine strategies in DLBCL have been limited to targeting surface antigens of mature B cells with antibodies (2), antibody–drug conjugates (3), or chimeric antigen receptor–equipped T cells (4). Disappointments in the efficacy of small-molecule inhibitors targeting oncogenic B-cell receptor signaling arise from the extraordinary clinical, biological, and genetic heterogeneity of DLBCL, resulting in short-lived responses and inadequate patient stratification (5, 6). A better molecular understanding of the oncogenic mechanisms in DLBCL is warranted for rational personalized medicine approaches to emerge. Hence, functional characterization of the genomic drivers in DLBCL is critically needed.

B-cell antigen receptor (BCR) conveys signals that determine the expansion, differentiation, and survival of maturing B cells in the germinal center (GC) reaction (7, 8). In B-cell lymphomas, malignant cells have hijacked pathways that originate from the BCR through somatic mutations and maintain a proliferating phenotype through transcriptional dysregulation and differentiation block (9). In activated B-cell (ABC)–like DLBCL, such genetic alterations cause chronic BCR signaling and activation of nuclear factor kappa B (NF-κB; refs. 10, 11). In addition, stereotypic usage of VDJ-recombined immunoglobulin gene segments gives BCRs capable of binding self-antigens (12). Moreover, antigen-independent tonic mode of BCR signaling is required for the survival of B cells and repurposed for oncogenic growth in germinal center B-cell (GCB) DLBCL (13, 14). As such, many lymphomagenic mutations mechanistically potentiate these modes of BCR signaling by increasing BCR levels on the cell surface (10), activating converging and downstream pathways (15–18), or disrupting negative feedback loops of the signaling (19, 20).

The germinal center regulator of B-cell maturation Kelch-like family member 6 (*KLHL6*) is recurrently mutated in DLBCL, but a potential link to oncogenic BCR signaling has remained elusive. KLHL6 belongs to the bric-a-brac tramtrack broad (BTB)-Kelch family of proteins, which function as exchangeable substrate adapters for Cullin-3-RING-ligase (CRL) E3 ubiquitination machinery targeting proteins for degradation. *KLHL6* was first cloned from ovine Peyer’s patch tissues, and its expression is restricted to human GC B cells and most GC-originating B-cell lymphomas (21, 22). The importance of KLHL6 expression for B-cell function was demonstrated *in vivo* and *in vitro*, as *Klhl6* knockout mice had severely reduced mature B-cell numbers, impaired GC formation following ovalbumin immunization and had blunted responses to BCR stimulation *in vitro* (23). However, the indispensable role of KLHL6 in the GC reaction is controversial, and a potential link with BCR signaling lacks mechanistic evidence (24).

Somatic mutations in the N-terminal BTB domain of *KLHL6* have been reported in 1% to 10% of DLBCLs (25–27) and 1% to 3% of chronic lymphocytic leukemia (CLL; refs. 28, 29). In DLBCL, *KLHL6* mutations have neither been linked to any molecular subtypes nor are they involved in emerging genomic taxonomies (30, 31). More recently, BTB domain mutations have been found in autoreactive B-cell clones responsible for autoantibody production in cold agglutinin disease (32). First efforts to understand the consequences of these mutations have linked the loss of KLHL6 with NF-κB activation through stabilization of RNA decay factor Roquin-2, which leads to downregulation of the negative regulator A20 (*TNFAIP3*; ref. 33). Some BTB domain mutations were suggested to result in impaired Cullin-3 binding proposing a loss-of-function. However, these first studies have relied on protein interaction discovery in cell lines that do not recapitulate the molecular context of GC-derived B-cell lymphomas. Thus, how *KLHL6* mutations promote lymphomagenesis remains incompletely characterized, impeding rational therapeutic targeting of these alterations.

To determine the phenotypic consequences of various *KLHL6* gene mutations found in DLBCL, we examined the molecular portrait of KLHL6 in DLBCL and applied high-throughput proteomic profiling and functional characterization to discover how these mutations alter BCR expression and function. We found that KLHL6 interacts with the BCR and regulates its surface levels. Notably, mutations in the BTB domain led to increased BCR levels and signaling, thereby adding to the multitude of targetable mechanisms of oncogenic BCR signaling in B-cell malignancies.

## Results

### Physiologic KLHL6 Protein Expression Pattern Is Frequently Absent in MCD/C5-Like ABC DLBCLs

**Figure 1.**
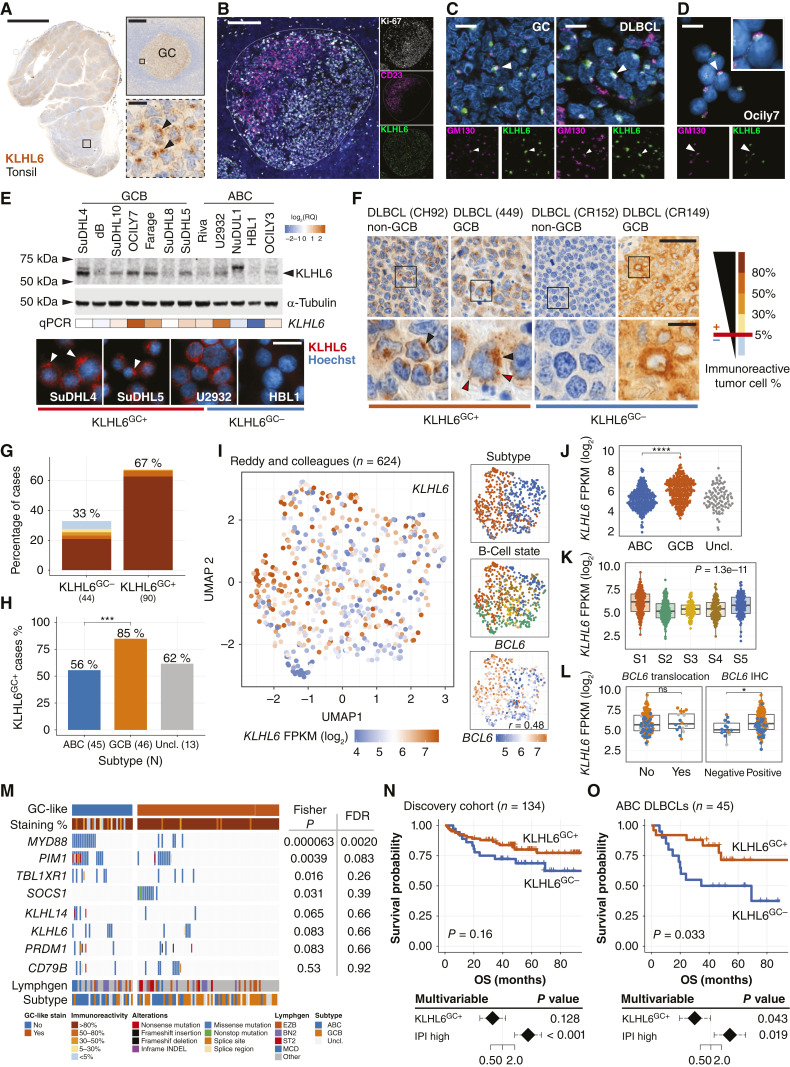
Molecular framework and clinical significance of KLHL6 protein expression in DLBCL. *P* values for pairwise comparisons indicated as >0.05, ns; <0.05, *; <0.01, **; <0.001, ***; <0.0001, ****. **A,** Representative microscopy images of IHC staining of the human tonsil for KLHL6. Arrowheads indicate cells with representative GC-like subcellular staining (KLHL6^GC+^). Scale bars, 5,000, 200, and 20 μm. **B,** Representative IF microscopy image of a GC in a reactive human tonsil (tonsils, *n* = 3; lymph nodes, *n* = 4) co-stained for light zone marker CD23, dark zone marker Ki-67, and KLHL6. Scale bar, 200 μm. **C** and **D,** IF microscopy images representative of KLHL6 subcellular localization, co-stained with GM130 (Golgi marker) and DAPI (DNA marker, blue) obtained from GC of lymph node and DLBCL tissue at 40× with optical sectioning and Ocily7 cells at 100× with conventional IF microscopy (**D**). White arrowheads pinpoint examples of colocalization. Scale bar, 10 μm. **E,** KLHL6 expression in DLBCL cell lines. Top, Western blot lanes with arrowhead showing the size of overexpressed KLHL6 construct (lane not shown). Middle, Heatmap of *KLHL6* RNA transcript levels by qPCR. Bottom, Representative IF microscopy images of endogenous KLHL6 expression. White arrowheads pinpoint KLHL6^GC+^ expression. RQ, relative quantification. Scale bar, 20 μm. **F** and **G,** Representative KLHL6 IHC staining patterns in four tumors (**F**, training set, *n* = 39) and quantification in the discovery cohort (**G**, *n* = 134). Black arrowheads denote KLHL6^GC+^, whereas red arrowheads show additional dispersed puncta. Interpretation of KLHL6 GC-like staining shown below the images. On the right, a scale to quantify KLHL6 immunoreactivity. GC-like pattern with immunoreactivity ≥5% was considered positive. Scale bars, 50 and 10 μm. **H,** Bar plot of KLHL6^GC+^ cases in the discovery cohort (*n* = 134) according to molecular subtype. Fisher exact test comparing ABC and GCB. **I–L,** UMAP dimensionality reduction of 624 DLBCLs (34) with RNA sequencing expression data colored according to KLHL6 expression. Next to the plot, inlets of UMAP projections and dot and box plots showing KLHL6 expression within (**J**) molecular subtypes, (**K**) B-cell states and (**L**) BCL6 expression, translocations, and IHC positivity. Mann–Whitney *U* test comparisons in **J** and **L**; Kruskal–Wallis in **K**. **M,** Oncoprint of mutations in driver genes most significantly associated with KLHL6^GC^ status in the discovery cohort (*n* = 110). Select MCD drivers and *KLHL6* shown, genes mutated in ≥5% of the cases considered, multiple testing correction performed with FDR. **N–O,** Kaplan–Meier survival estimates for overall survival (OS) according to KLHL6^GC+^ pattern. Log-rank test. Below the estimates: multivariable Cox regression model’s HRs, 95% confidence intervals, and *P* values for OS with KLHL6^GC+^ and IPI.

To get an overview of KLHL6 protein expression in DLBCL tissues, we first performed immunostaining of tissue sections from reactive lymphoid specimen and diagnostic DLBCL samples. In lymphoid tissues, KLHL6 protein expression was mainly constrained to GC B cells that were not spatially restricted to either light zone (LZ) or dark zone (DZ) of the GC (Fig. 1A and B; Supplementary Fig. S1A and S1B). At the single-cell level, *KLHL6* expression varied across GC B-cell subpopulations with the highest expression in transitional B cells whereas cells undergoing DZ and memory B-cell differentiation had downregulated *KLHL6* (Supplementary Fig. S1C). Colocalization studies demonstrated that KLHL6 localized on perinuclear vesicles concentrated in the immediate vicinity of the Golgi apparatus (Fig. 1C and D; Supplementary Fig. S1D and S1E). This physiologic GC-like KLHL6 expression pattern (KLHL6^GC+^) was evident in most malignant cells in the examined DLBCL tissues and cell lines (Fig. 1C–F). In a subset of DLBCL cases and cell lines, however, malignant cells did not express KLHL6 or they exhibited a varying degree of cytosolic protein expression pattern (KLHL6^GC–^; Fig. 1E and F).

In the discovery cohort of 134 primary DLBCLs, the overall immunoreactivity for KLHL6 antibody was high, with the KLHL6^GC+^ phenotype present in 67% of the samples (Fig. 1G; Supplementary Fig. S1F; Supplementary Table S1A). KLHL6^GC+^ was strongly enriched in the GCB subtype, whereas 44% of the ABC DLBCLs were KLHL6^GC–^ (Fig. 1H). At the mRNA level in an expanded data set of 624 DLBCLs (34), *KLHL6* gene expression was significantly higher in GCB DLBCLs compared with ABC DLBCLs (Fig. 1I and J). In detail, *KLHL6* expression was the highest in the lymphomas dominated by B cells of S1 state, corresponding to GC B cells (Fig. 1I–K; ref. 35), 90% of which were deemed KLHL6^GC+^ on the protein level. *KLHL6* gene expression correlated with the expression of other GCB markers including *BCL6* and *MEF2B* and 90% (70 of 78) of the KLHL6^GC+^ were also BCL6+ by IHC (Fig. 1L; Supplementary Table S1B and S1C). Of note, neither elevated *KLHL6* (3q27.1) gene expression nor KLHL6^GC+^ phenotype was associated with *BCL6* (3q27.3) translocations (Fig. 1L; Supplementary Table S1C).

To further characterize the subset of lymphomas that were KLHL6^GC−^, we expanded our analysis to the genomic level. We found that somatic mutations in *MYD88* and other ABC drivers linked to the MCD/C5 genotype were associated with KLHL6^GC−^ (Fig. 1M; Supplementary Fig. S1G). Besides aberrations characteristic of MCD/C5 subtype, we observed that *KLHL6* mutations itself more common with KLHL6^GC−^ phenotype (Fig. 1M; Supplementary Fig. S1G). Clinically, KLHL6^GC+^ did not associate with underlying patient characteristics besides cell of origin (Supplementary Table S1D). In contrast, although there was a trend toward poor survival for KLHL6^GC−^ in the discovery cohort (Fig. 1N), KLHL6^GC−^ phenotype translated to poor survival in the ABC DLBCL subtype independently of the International Prognostic Index (IPI; Fig. 1O). Collectively, these findings show that KLHL6^GC+^ is characteristic of GCB DLBCLs, whereas this phenotype is absent in a subset of ABC DLBCLs with MCD/C5-like features and poor outcome.

### BTB and Kelch Domain Mutants Correspond to KLHL6^GC–^ and KLHL6^GC+^ Subcellular Expression Patterns

**Figure 2.**
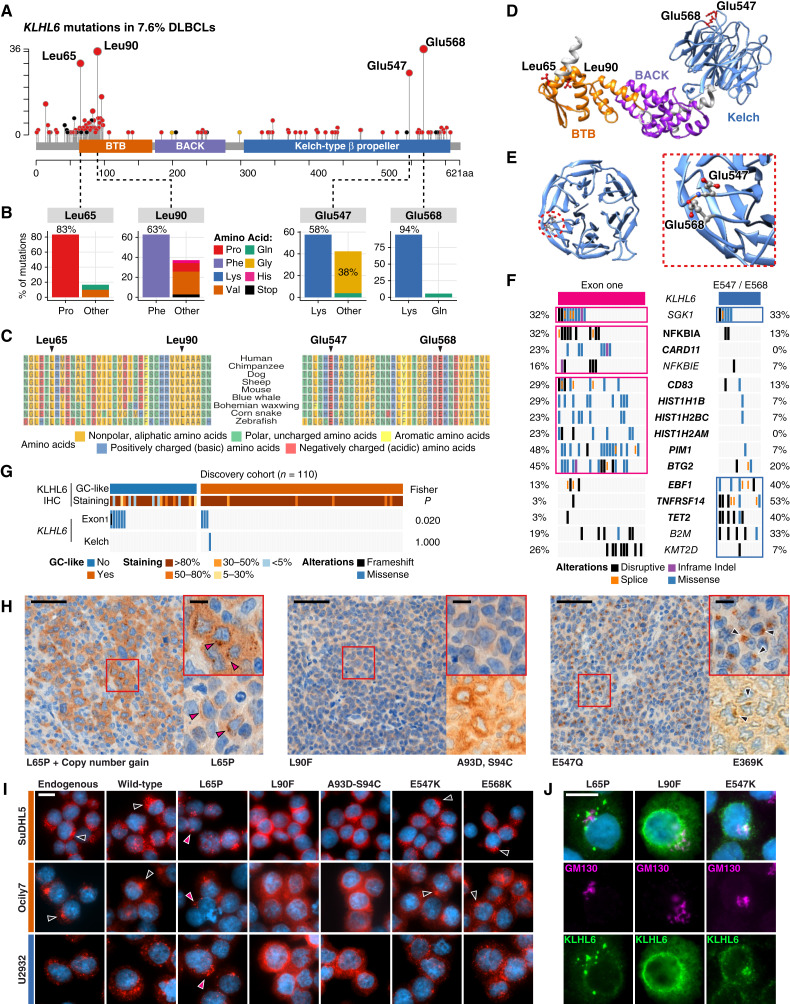
Recurrent *KLHL6* mutations and their impact on subcellular localization in lymphoma cells. **A,** Lollipop plot of *KLHL6* mutations reported in 303 DLBCL tumors. **B,** Bar plots showing the fraction of different amino acid (AA) changes according to the most recurrently mutated AA residues in DLBCLs. Most common AA replacement per residue highlighted as the left hand side bar. **C,** Evolutionary conservation of KLHL6 AA sequences in the vicinity of the most frequently mutated AA residues across selected species. **D,** Predicted three-dimensional (3D) structure of KLHL6 according to *in silico* modeling using AlphaFold. Protein domains are shown with different color shading, and recurrently mutated AA residues are highlighted. **E,** 3D model of KLHL6 Kelch domain seen from above with recurrently mutated glutamic acid residues E547 and E568 shown. Inlet highlights colocalization. **F** and **G,** Genetic (**F**) and protein localization (**G**) features differentially associated with *KLHL6* mutations in exon one (encoding the BTB hotspot) and Kelch domain. **F,** Mutations enriched in other genes co-occurring with different types of *KLHL6* mutations in the Lacy and colleagues data set (*n* = 928; ref. 38). Genes mutated in ≥5% of the patients included in the analysis. Boxed rows indicate enriched genomic drivers (Fisher exact < 0.05), genes with FDR < 0.1 bolded. **G,** Co-occurrence with KLHL6 staining patterns according to KLHL6^GC+^ status in the discovery cohort (*n* = 110). **H,** Light microscopy images of diagnostic DLBCL tissue sections immunohistochemically stained for KLHL6 from patients with different *KLHL6* mutations according to the original variant calls or re-analysis. Annotated AA changes of mutations highlighted per representative figure. Red arrowheads indicate aberrant puncta in tissues with *KLHL6*^L65P^ mutation. Black arrowheads indicate KLHL6^GC+^ pattern. Scale bars, 50 μm (10 μm for inlets). **I,** Representative IF microscopy images of GCB cell lines SuDHL5 and Ocily7 and ABC cell line U2932 with ectopic expression of KLHL6 WT or mutants indicated on the top. Black arrowheads indicate representative KLHL6^GC+^ patterns and red arrowheads indicate aberrant vesicles of *KLHL6*^L65P^. Scale bar, 10 μm. **J,** Representative IF microscopy images of SuDHL5 cells expressing *KLHL6*^L65P^, *KLHL6^L90F^*, or *KLHL6*^E547K^ mutants. Cells co-stained for KLHL6 and Golgi marker GM130. Co-stainings of other mutants shown in Supplementary Fig. S2E. Scale bar, 10 μm.

Pooled analysis of the reported *KLHL6* mutations in DLBCLs (7.6%, 303/4,011 patients; Supplementary Table S2A and S2B) revealed that the mutations were mostly of missense type (89%) and four recurrent amino acid residues accounting for 33% of the reported mutations (Fig. 2A–C). As established, the N-terminal BTB domain was targeted by recurrent point mutations most commonly affecting leucine 65 and leucine 90 with proline (KLHL6^L65P^) or phenylalanine (KLHL6^L90F^) substitutions, respectively (Fig. 2B). Additionally, we identified two recurrently mutated residues in the C-terminus of KLHL6 in the Kelch domain (Fig. 2A). These Kelch mutations switched evolutionary conserved negatively charged glutamic acid residues (E547 and E568) to mostly positively charged lysines (KLHL6^E547K^ and KLHL6^E568K^; Fig. 2B and C). *In silico* structural modeling (36) revealed that these residues were situated side-by-side on the outer surface of the Kelch domain, lining the putative substrate–binding surface of the domain (Fig. 2D and E). Interestingly, these C-terminal mutations have not been reported in CLL (37) with 2.8% *KLHL6* mutation rate, solely relating to the N-terminus (Supplementary Fig. S2A). In 928 DLBCLs (38), mutations in *SGK1* were enriched in patients with either N- or C-terminal mutations, however, select NF-κB pathway genes and hypermutable targets were enriched in cases with exon 1 mutations encoding the BTB domain mutation hotspots of *KLHL6*, whereas mutations in *TNFRSF14*, *TET2*, and *EBF1* were enriched in cases with Kelch domain mutations (Fig. 2F; Supplementary Fig. S2B).

Next, we characterized KLHL6 mutants for subcellular protein expression pattern in the available DLBCL samples. In the discovery set, we found that the association between the loss of KLHL6^GC+^ localization pattern and *KLHL6* mutations was limited to the BTB domain compromising N-terminal mutations, whereas Kelch domain affecting mutations retained physiologic attenuation (Fig. 2G and H; Supplementary Fig. S2C). Moreover, KLHL6^L65P^ localized in cytosolic aberrant vesicles in the DLBCL cells, whereas in a patient with KLHL6^L90F^ in the original dataset KLHL6 was expressed throughout the cytosol (Fig. 2H). Notably, a similar cytosolic pattern was seen in one DLBCL sample from our training set which upon genomic interrogation of *KLHL6* exon one carried a double mutation of *KLHL6* (KLHL6^A93D-S94C^; Fig. 2H).

Introduction of wild-type (WT) and different KLHL6 mutants to GCB and ABC DLBCL cell lines with endogenous KLHL6 expression recapitulated the findings from DLBCL samples and confirmed that BTB domain mutants were localized in nonphysiologic cytosolic patterns (Fig. 2I and J; Supplementary Fig. S2D). By contrast, WT and Kelch domain mutant KLHL6 displayed the GC-like pattern (KLHL6^GC+^, Fig. 2I and J; Supplementary Fig. S2E). Furthermore, ectopic BTB domain mutants abolished the physiologic expression pattern of endogenous KLHL6 to a varying degree, suggesting that these mutations could possess dominant capabilities over the endogenous protein (Fig. 2I and J). Together, these findings reveal a dichotomous pattern of mutations targeting the BTB and Kelch domains of KLHL6 in DLBCLs that disrupt subcellular localization in malignant B cells.

### BTB Domain Mutations Disrupt Heterodimerization of Cullin-3-Ring-Ligase Substrate Adapter KLHL6

**Figure 3.**
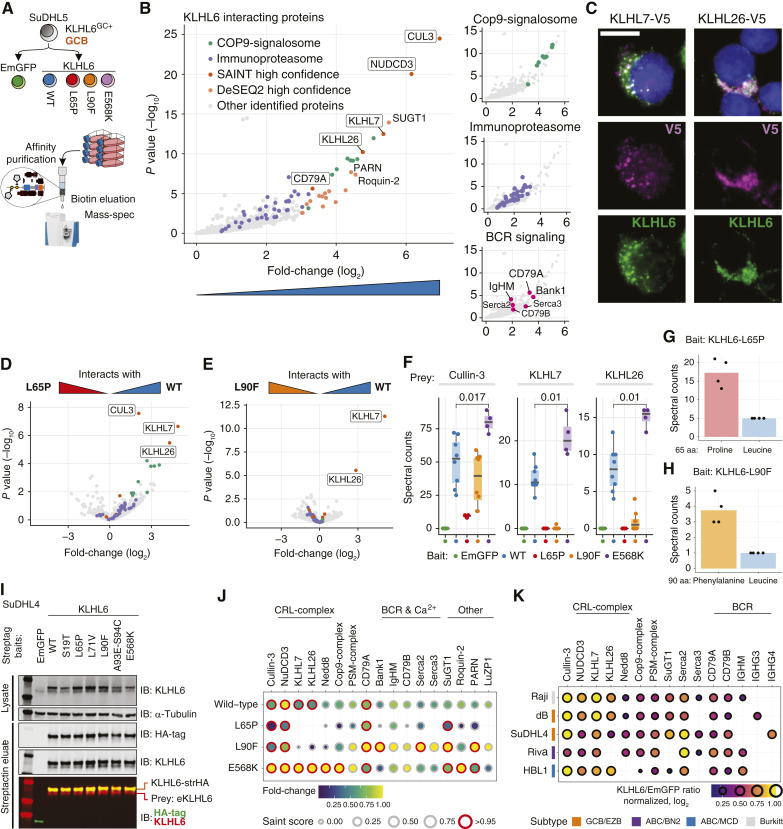
KLHL6 interacts with BCR and forms homodimers and heterodimers that are differentially impacted by recurrent mutations. **A,** High-throughput proteomic approach to determine KLHL6 protein interactions and impact of recurrent mutations in DLBCL context. C-terminally tagged KLHL6 constructs encoding WT protein and different mutants or EmGFP (tagged and nontagged) controls were used in 4–8 biological replicate purifications in two batches of mass spectrometry. **B,** Volcano plot of DESeq2 model between WT KLHL6 and EmGFP pull-downs showing only the KLHL6 interacting proteins (positive fold-change). Labeled gene names of proteins denote high-confidence interactors detected by SAINT analysis. Plots on the right highlight interacting protein components of the Cop9-signalosome, immune proteasome, and BCR signaling. **C,** Representative IF microscopy images showing colocalization between endogenous KLHL6 and C-terminally V5-tagged KLHL7 and KLHL26 constructs. Scale bar, 10 µm. **D** and **E,** Volcano plots of differentially interacting proteins between WT KLHL6 (eight replicates) and (**D**) KLHL6^L65P^ (four replicates) and (**E**) KLHL6^L90F^ (eight replicates). Proteins with positive fold-change (log_2_) interact more strongly with the WT bait. **F,** Box plots of spectral counts of peptides from selected prey proteins in the pull-downs according to different baits. Dots represent biological replicates. Mann–Whitney *U* test for comparisons, *P* values shown. **G** and **H,** Bar and dot plots of spectral counts for specific peptides in BTB mutant KLHL6 pull-downs encoding either mutated or WT amino acids from (**G**) *KLHL6*^L65P^ and (**H**) *KLHL6*^L90F^ peptides. Dots represent individual replicates, and bars represent their means. All analyzed pull-downs contained mutant (bait) and WT (prey) peptides. **I,** Western blot analysis of SuDHL4 cells transduced with Strep-tagged WT and mutant KLHL6 constructs or EmGFP control. In the eluate, purified for Strep-tag, one protein (bait) is detected with HA-antibody, whereas two proteins (bait and prey) are detected with KLHL6 antibody confirming homodimerization. **J,** Balloon plot showing the interactors of WT and mutant KLHL6 baits according to SAINT analysis. **K,** Balloon plot of WT KLHL6 interactors compared with EmGFP control purifications identified with AP-MS across multiple lymphoma cell lines from different molecular backgrounds. Validated key interactors identified from SuDHL5 are shown.

To uncover B-cell pathways affected by *KLHL6* mutations, we introduced C-terminally tagged KLHL6 constructs to SuDHL5 GCB cells with high *KLHL6* gene expression and KLHL6^GC+^ phenotype, affinity-purified the lysates for the baits with their bound complexes and identified the interacting proteins with high-throughput mass spectrometry (AP-MS, Fig. 3A; Supplementary Table S3A–G).

The protein–protein interactome of WT KLHL6 reflected its role as a CRL substrate adapter for ubiquitination and subsequent proteasomal degradation of target proteins (Fig. 3B). These interactors included Cullin-3, Kelch-chaperone NuDCD3 (39), the CRL deneddylating Cop9-signalosome complex, and immunoproteasome components. Additionally, we identified proteins involved in BCR signaling of which subunit CD79A and the adapter protein BANK1 were detected with the highest confidence (Fig. 3B). Furthermore, we recorded interactions with SUGT1, involved in membrane trafficking of MHC I and II complexes in B cells (40), and a previously reported interactor Roquin-2 (33). Heterotypic interactions with other BTB-Kelch family proteins KLHL7 and KLHL26 proposed that KLHL6 forms heterodimeric configurations characteristic to BTB-Kelch family members. Notably, KLHL7 and KLHL26 have been shown not to dimerize with each other (41), and we found that these proteins were expressed in different subcellular compartments of DLBCL cells and colocalized with endogenous KLHL6 (Fig. 3C). While KLHL7 and KLHL6 proteins colocalized in perinuclear vesicles, the colocalization with KLHL26 was more diffusely present in perinuclear membranous structures.

To delineate mutation-dependent changes in KLHL6 protein interactions, we compared the proteomic data between WT and mutant KLHL6 baits. All baits were ubiquitinated in multiple residues and most of the protein–protein interactions were retained by the mutant baits, suggesting that they remained involved in CRL activities (Supplementary Table S3A–G). Strikingly, the BTB domain mutations L65P and L90F ablated heterodimerization of KLHL6 with KLHL7 and KLHL26 (Fig. 3D–F). In contrast, the Kelch-domain variant KLHL6^E568K^ bait was more tightly engaged by these BTB-Kelch family proteins (Fig. 3F). Conflicting with a previous report, we found that Cullin-3 co-purified with all the baits in every replicate, albeit to a lesser degree with KLHL6^L65P^ (Fig. 3F). These patterns prompted us to ask whether BTB mutant KLHL6-baits still formed CRL complexes through homodimerization with endogenous KLHL6. We recovered WT and mutant KLHL6 peptide spectra from AP-MS data of both BTB-mutant baits, strongly arguing in favor of homodimerization with endogenous KLHL6 (Fig. 3G and H). Additionally, affinity purifications (APs) with several KLHL6 WT and patient-derived mutant baits co-purified endogenous KLHL6, thus confirming the formation of a homodimer CRL that was not disrupted by the examined mutations (Fig. 3I).

Given that KLHL6 mutants could form homodimer CRLs, we asked how mutant KLHL6 baits would affect the KLHL6^WT^ interactome (Fig. 3J). A stronger association between KLHL6^E568K^ and CRL components, the Cop9-signalosome, and CRL-activating posttranslational modification Nedd8 suggested that this Kelch variant could be involved in constitutively active forms of CRL complexes. However, KLHL6^E568K^ did not differ in the interaction with CD79A and BANK1, in contrast to BTB mutant KLHL6^L90F^ (Fig. 3J).

Finally, to determine which KLHL6 interactions were shared by diverse molecular contexts of B-NHL, we examined the KLHL6 interactome in a broader panel of DLBCL cell lines. Most of the CRL components and many other interactors were confirmed throughout the cell line panel and, notably, all the pull-downs confirmed interactions with the BCR, including both IgM and IgG isotypes (Fig. 3K; Supplementary Fig. S3A and S3B; Supplementary Table S3H and S3I). Altogether, these experiments indicate that KLHL6 forms homodimers and heterodimers and interacts with the CRL machinery and BCR components. Specifically, recurrent BTB mutations compromise the heterodimeric constellations.

### Kelch Domain–Linked Proximity–Dependent Labeling Reveals the Substrate Map of KLHL6

**Figure 4.**
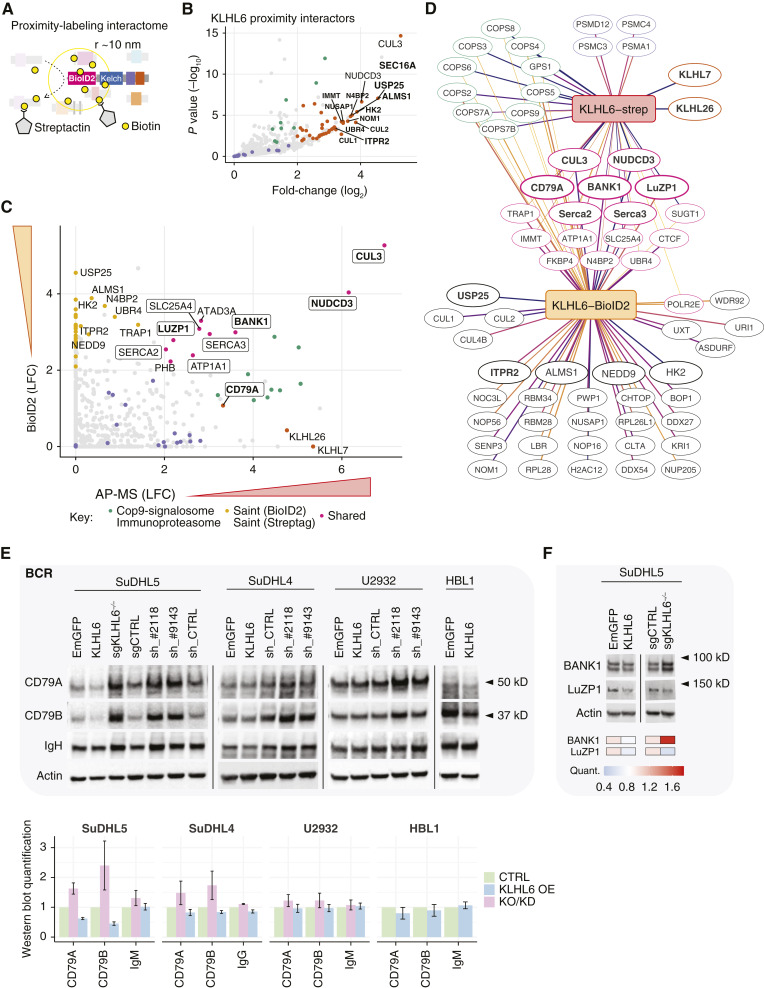
Complementary interactome screening with Kelch-domain coupled proximity-labeling reveals potential substrates of KLHL6 in B-cell context. **A,** Schematic representation of proximity-labeling (PL) substrate screening with BioID2 approach for C-terminally tagged KLHL6. **B,** Volcano plot of the DESeq2 model showing the differentially interacting proteins between BioID2-tagged WT KLHL6 and EmGFP constructs showing only the KLHL6-interacting proteins with a positive log_2_-transformed fold-change (LFC). Colors as in Fig. 3B. **C,** Comparison of KLHL6 interactomes obtained with the complementary protein interaction screening methods. Evidence for protein binding by APs (“AP-MS”) and proximity-labeling (“BioID2”) are indicated with LFCs of DESeq2 models with respective EmGFP control pull-downs. Labels indicate proteins with strong evidence by both methods and AP/PL-specific interactors not considered common contaminants in mass spectrometry experiments. **D,** Summary of identified KLHL6 interactors with the complementary screening methods. **E,** Western blots showing the impact of KLHL6 expression manipulation at the protein levels of BCR components CD79A, CD79B, and IgH (IgM or IgG) in four different cell lines. Below, bar blots show the quantified protein levels of the BCR components in cells with ectopic KLHL6 overexpression (KLHL6 OE) or KLHL6 silencing (KO, knockout; KD, knockdown) normalized to the total protein levels in relation to their respective controls (EmGFP, sgCTRL, or sh_CTRL). **F,** Western blots showing the impact of KLHL6 expression manipulation on the protein levels of its proximity interactors BANK1 and LuZP1. Heatmaps show the average quantified protein levels from KLHL6 overexpressing or knockout cells normalized to β-actin in relation to the EmGFP- or sgCTRL-transduced cells.

Apart from the protein components that form the KLHL6-CRL machineries, their interactions with the substrates they process are likely more transient and, thus, less likely to be detected by purification of protein complexes. Therefore, to obtain a molecular map of potential substrates recognized by KLHL6, we complemented our AP-MS interactome screen with KLHL6 baits fused with a Kelch domain adjacent proximity–dependent biotin ligase (BioID2; Fig. 4A; ref. 42).

We uncovered additional KLHL6 interactors with high confidence and validated many of the AP-MS interactors with BioID2 (Fig. 4B–D; Supplementary Table S4A–C). Novel interactors included ubiquitin turnover–related proteins such as deubiquitinating enzyme 25 (USP25) and E3 ubiquitin ligase UBR4 (Fig. 4B). We found further support for the localization of KLHL6 in endoplasmic reticulum (ER) and protein cargo–related compartments with high-confidence interactors, such as the Serca-type Ca^2+^ pumps, ER exit site marker SEC16A, and BANK1-associated Ca^2+^ channel inositol 1,4,5-trisphosphate (IP3) receptor type 2 (ITPR2; Fig. 4B; Supplementary Fig. S4A; ref. 43).

By contrast, some KLHL6 AP-MS interactors were poorly captured by BioID2. Among these, the N-terminal BTB-mediated heterodimeric interactions with KLHL7 and KLHL26 were poorly evidenced as labeling distance and protein stoichiometry limit the detection of interactors with C-terminal proximity labeling. By combining the data from the AP-MS and BioID2 interaction screens, we revealed that besides CRL components and electrolyte pumps, BCR signaling molecules CD79A and BANK1, together with LuZP1, a centrosomal actin modulator involved in ciliogenesis (44, 45), were captured by both affinity and proximity interactome screening methods, strongly suggesting that these proteins represented substrates of the KLHL6-CRL (Fig. 4C and D).

Next, we asked whether manipulation of KLHL6 expression impacted the protein levels of these interactors. In the examined KLHL6^GC+^ GCB cell lines, overexpression of KLHL6 WT downregulated the protein levels of BCR components CD79A and CD79B, whereas CRISPR-Cas9 or RNAi-mediated silencing increased their levels (Fig. 4E). By contrast, the impact on immunoglobulin heavy chain (IgH) levels was not evident (Fig. 4E). In the ABC cell lines U2932 and HBL1, however, the impact of KLHL6 manipulation was only modest on the expression levels of BCR components (Fig. 4E). To clarify these observations, we examined the KLHL6-BioID2 interactome in U2932 and validated BANK1 and LuZP1 interactions, whereas support for BCR components was minimal (Supplementary Fig. S4B; Supplementary Table S4D). BANK1 and LuZP1 levels were also downregulated in response to KLHL6 overexpression (Fig. 4F; Supplementary Fig. S4C). Together, these experiments suggest that KLHL6 targets BCR components, BANK1, and LuZP1 proteins for degradation in the GCB context.

### KLHL6 Mutants Differentially Perturb Antigen Receptor Surface Levels and Signaling Responses

**Figure 5.**
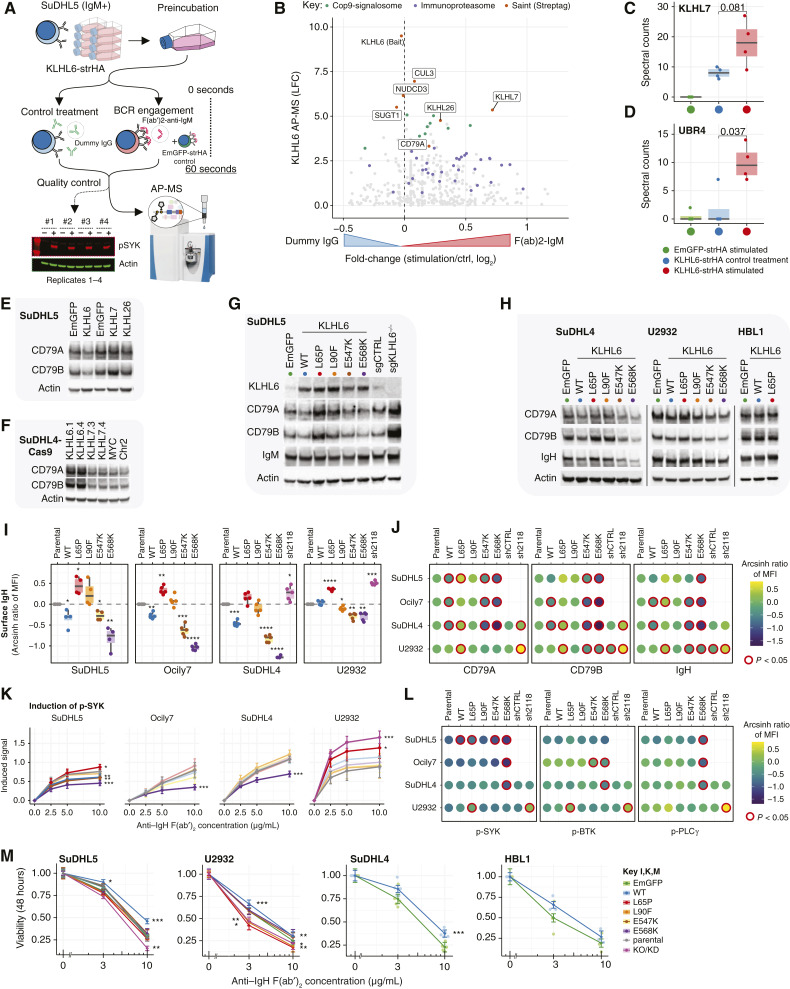
Recurrent KLHL6 mutations alter levels of BCR components and impact responses to BCR stimulation. **A,** Strategy to investigate KLHL6 interactome upon BCR engagement. SuDHL5 cells with ectopic expression of Strep-tagged KLHL6 bait were treated at RT for 60 seconds with anti–human IgM F(ab′)_2_ fragment or nonspecific goat IgG antibody. Snap-frozen lysates were affinity-purified, and the interacting proteins were identified with mass spectrometry (AP-MS). SuDHL5 cells expressing Strep-tagged EmGFP were stimulated and analyzed as a control by AP-MS. **B,** Differences in KLHL6 interactome between the conditions. *Y* axis represents the LFC of KLHL6 interacting proteins against EmGFP control purifications in the DESeq2 model from Fig. 3B. **C** and **D,** Box and dot plots of non-normalized spectral counts of (**C**) KLHL7 and (**D**) UBR4 with induced interaction with KLHL6 bait upon stimulation. *P* values calculated with the Mann–Whitney *U* test. **E** and **H,** Western blot analyses of CD79A, CD79B, and IgH (IgM or IgG) in (**E**) SuDHL5 cells transduced with EmGFP control and nontagged KLHL6 WT or V5-tagged KLHL7 and KLHL26 overexpression constructs (**F**) Cas9-expressing SuDHL4 cells transduced with KLHL6 (KLHL6.1 and KLHL6.4) or KLHL7 (KLHL7.3 and KLHL7.4) targeting sgRNAs or control sgRNAs (MYC and Chr2; **G**) SuDHL5 cells transduced with KLHL6 WT and mutant constructs and (**H**) SuDHL4, U2932, and HBL1 cell lines transduced with WT and mutant KLHL6 constructs. **I** and **J,** Impacts of different KLHL6 constructs on the surface levels of all BCR components in SuDHL5 (*n* = 4), Ocily7, SuDHL4, and U2932 (*n* = 5) cell lines measured with flow cytometry. Values on *y*-axis (**I**) and the color fill (**J**) represent arcsinh ratio of MFI normalized to expression in parental cells. *P* values calculated with two-sided one-sample *t* test and (**J**) corrected for multiple testing with the Bonferroni method. **K,** Induction of SYK kinase phosphorylation measured by phospho-flow in various lymphoma cells expressing indicated KLHL6 mutants with different anti–IgH F(ab′)_2_ concentrations. Data pooled from 5 to 6 independent experiments. *P* values were calculated with one-way ANOVA followed by the Dunnett multiple comparisons test against the unstimulated control. **L,** Balloon plot of anti–IgM-induced or anti–IgG-induced (10 µg/mL) signaling in parental and genetically modified SUDHL5, Ocily7, SuDHL4, and U2932 cells overexpressing KLHL6 WT and mutant constructs measured by phospho-flow. Color fill shows the mean phosphorylated levels of SYK, BTK, and PLCγ as arcsinh ratio of MFI normalized to expression unstimulated cells. Statistical significance according to the one-way ANOVA test followed by the Dunnett multiple comparisons test. **M,** Cell viability (Cell titer Glo) in various cell lines with different KLHL6 genetic manipulations after 48 hours of treatment in indicated concentrations of F(ab′)_2_ anti-IgM or F(ab′)_2_ anti-IgG. Viability normalized to control treatment with unspecific goat IgG antibody. *P* values are calculated using the two-sided *t* test against EmGFP-expressing cells.

To examine the functional connection between KLHL6 and BCR signaling, we tested if KLHL6 interactions were altered upon BCR activation by performing AP-MS for KLHL6 bait at 60 seconds after BCR cross-linking in SuDHL5 cells (Fig. 5A; Supplementary Fig. S5A and S5B). We observed that although the protein complex of KLHL6 remained mostly unchanged, the heterodimerization with KLHL7 was strengthened and the interactions with UBR4 and immunoproteasome were induced (Fig. 5B–D; Supplementary Fig. S5C and S5D). However, in contrast to the manipulation of KLHL6, overexpression or CRISPR-Cas9 silencing of KLHL7 did not similarly alter the levels of BCR components in cell lysates suggesting that KLHL6-KLHL7 heterodimerization was dispensable for BCR downregulation (Fig. 5E and F; Supplementary Fig. S5E–G).

Given that the KLHL6 mutants retained homodimerization, however, we proceeded to characterize the consequences of different KLHL6 mutants for BCR expression and signaling in DLBCL cells. Analysis of total and surface protein levels of CD79A, CD79B, and IgM/IgG in isogenic DLBCL cell lines with endogenous KLHL6 (parental), or overexpression of KLHL6^WT^ or recurrent mutants revealed differential impacts by the BTB and Kelch mutations on the BCR phenotype. Strikingly, the BTB domain mutants KLHL6^L65P^ and KLHL6^L90F^ failed to downregulate and even increased the levels of CD79A and CD79B across all tested cell lines (Fig. 5G and H; Supplementary Fig. S5H and S5I). In contrast, Kelch domain mutants KLHL6^E547K^ and KLHL6^E568K^ retained their ability to promote downregulation of these molecules also in the ABC-DLBCL cell line U2932, in which the overexpression of KLHL6^WT^ had little impact (Fig. 5H; Supplementary Fig. S5H and S5I). The changes in the total levels of CD79A and CD79B were recapitulated by similar changes in the surface levels, whereas the surface level of IgM/IgG (IgH) followed the changes in CD79A and CD79B (Fig. 5I and J; Supplementary Fig. S6), which is in agreement with the requirement of CD79A-CD79B heterodimer for IgH transportation to the plasma membrane (14). In detail, the cell surface levels of all BCR components CD79A, CD79B, and IgM were significantly decreased across the GCB cell lines following ectopic WT KLHL6 expression, whereas no such impact was seen in the ABC cell line U2932 (Fig. 5I and J). However, the overexpression of the Kelch domain mutants decreased the surface levels of BCR across all cell lines (Fig. 5I and J). In contrast, BTB mutants did not downregulate BCR surface levels, and KLHL6^L65P^ even increased the surface levels of all BCR components similarly to *KLHL6* silencing by short hairpin RNA (shRNA) in both ABC and GCB cell lines (Fig. 5I and J). These phenotypic differences in the surface levels of BCR components led us to hypothesize that BCR responses could differ between DLBCL cells expressing different KLHL6 mutants.

We engaged surface BCRs of isogenic DLBCL cells with endogenous levels or overexpressing WT or mutant KLHL6 proteins to profile changes in protein phosphorylation of intracellular proximal signaling kinases. In line with the lowest level of surface BCRs, overexpression of the Kelch domain mutant KLHL6^E568K^ attenuated the phosphorylation of proximal BCR-activated kinases such as Src family kinases (SFK, e.g., Lyn), SYK, BTK, and phospholipase C γ (PLC-γ) compared with the parental cells in GCB cell lines (Fig. 5K and L; Supplementary Fig. S7A). However, no significant change was observed in the ABC cell line U2932 with only a minor effect of KLHL6^E568K^ on the BCR surface levels (Fig. 5I–L). Overexpression of KLHL6 WT had a similar but less significant effect. The effects of Kelch domain mutants contrasted the overexpression of the BTB domain mutant KLHL6^L65P^, which led to significantly potentiated proximal BCR signaling responses in the GCB cell line SuDHL5 and the ABC cell line U2932 (Fig. 5L). In concordance with the surface expression levels, the shRNA knockdown of *KLHL6* (sh_#2118) mimicked that of KLHL6^L65P^ and led to significantly potentiated BCR signaling as compared with shRNA control cells (Fig. 5K and L; Supplementary Fig. S7A).

BCR engagement in the absence of a co-stimulatory signal induces apoptosis of B cells *in vitro* and the magnitude depends on intracellular Src kinase activity (46). Therefore, we hypothesized that manipulation of KLHL6 expression should alter these responses. When unchallenged, U2932 and SuDHL5 cells with various *KLHL6* manipulations grew at a similar rate, however, silencing of *KLHL6*-sensitized and WT overexpression desensitized lymphoma cells for activation-induced cell death (AICD; Fig. 5M; Supplementary Fig. S7B and S7C). Notably, in SuDHL5 cells, the BTB domain mutants did not compromise cell viability upon stimulation whereas these mutants sensitized U2932 cells to AICD similarly to KLHL6 knockdown (Fig. 5M). Despite the lower BCR expression levels in the Kelch domain mutant expressing cells, the viability was comparable with transduction controls and parental cell line (Fig. 5M).

**Figure 6.**
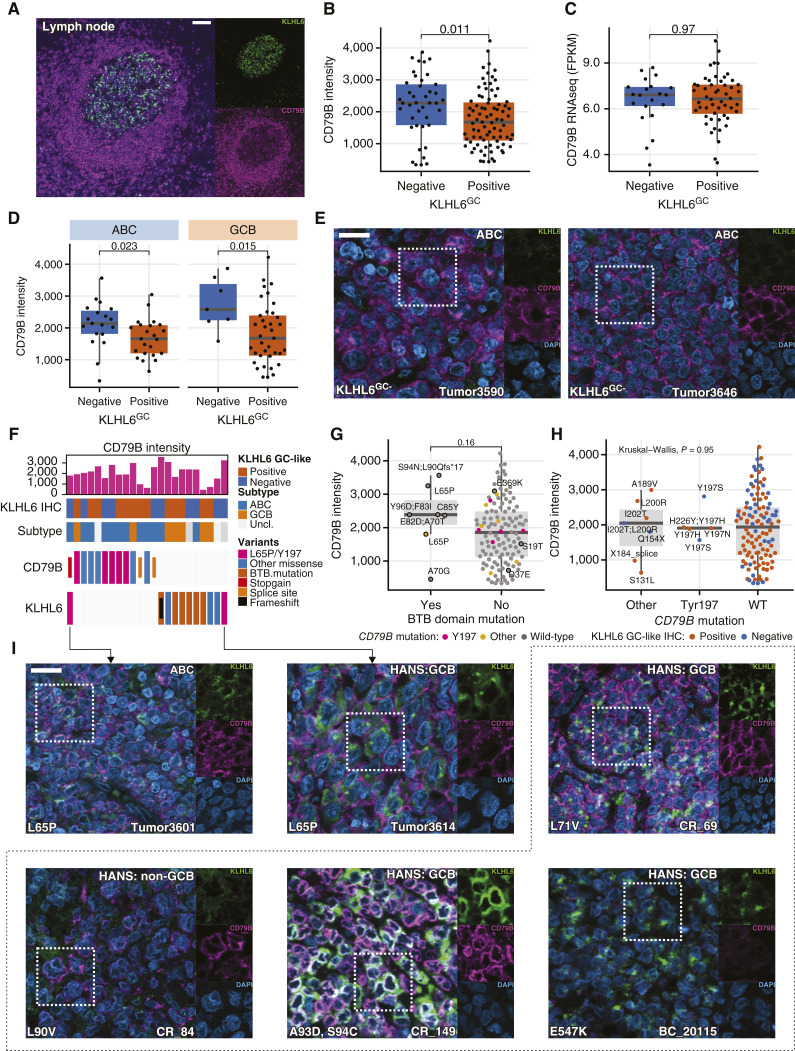
Interrogation of CD79B at the protein level in the discovery set and *KLHL6*-mutated tumors. *P* values for comparisons between two groups in box plots are calculated with the Mann–Whitney *U* test. Individual channels shown for the white dashed areas are shown right to the microscopy images. **A,** IF microscopy image of a GC in a reactive lymph node showing the highest CD79B intensity in the mantle zone, whereas KLHL6 positivity is restricted to the GC. Scale bar, 100 µm. **B** and **C,** Box and dot plots with *y*-axis showing (**B**) quantified CD79B intensity and (**C**) *CD79B* gene expression (from RNA sequencing) according to KLHL6^GC+^ IHC (*x*-axis). **D,** Box and dot plots of quantified CD79B intensity according KLHL6^GC+^ IHC phenotype within different molecular subtypes determined by gene expression profiling. **E,** Representative IF microscopy images of KLHL6^GC-^ tumors showing plasma membrane–like CD79B staining. Sections stained for CD79B, KLHL6, and DNA (DAPI) and images obtained with optical sectioning and 40× objective. Scale bar, 20 µm. **F,** Oncoprint of patients (columns) with *KLHL6* and/or *CD79B* mutations (*n* = 23). **G,** Box and dot plot of CD79B intensity according to *KLHL6* BTB domain encoding mutation status. Amino acid changes of all *KLHL6* mutations for available cases are annotated, and the presence of *CD79B* mutations is indicated with a color. **H,** Box and dot plot showing CD79B intensity according to *CD79B* mutations with Y197 hot spot considered separately. Color of the dots indicates KLHL6^GC^ IHC status and amino acid changes of *CD79B* mutations are annotated. **I,** Representative IF microscopy images of tumors with different *KLHL6* mutations co-stained for CD79B, KLHL6, and DNA (DAPI). Images within dashed area indicate additional *KLHL6*-mutated DLBCL tumors not included in the discovery cohort. Scale bar, 20 µm applies to all images.

Having established a functional link between KLHL6 and the BCR in cell lines, we re-examined the discovery cohort by immunofluorescent (IF) microscopy for KLHL6 and BCR marker (CD79B) at the protein level (Fig. 6A; Supplementary Fig. S8A–D). Quantification of the CD79B staining revealed that KLHL6^GC+^ tumors had lower CD79B levels than the tumors with KLHL6^GC-^ phenotype, whereas no such association was seen on *CD79B* gene expression (Fig. 6B and C; Supplementary Fig. S8E). The difference in CD79B protein was confirmed among the major subtypes, and *KLHL6* and *CD79B* mutations showed a mutually exclusive trend (Fig. 6D–F; Supplementary Fig. S8F). Notably, the tumors with *KLHL6* BTB mutations had a trend toward higher CD79B intensity, whereas *CD79B* mutations did not (Fig. 6G and H). In the tumors, *KLHL6*^L65P^ and *KLHL6*^L71V^ mutations were associated with membrane-like CD79B staining, whereas other BTB mutants had variable CD79B patterns (Fig. 6I; Supplementary Fig. S8G). Yet, the staining for CD79B was low in the tumor carrying *KLHL6*^E547K^ mutation (Fig. 6I; Supplementary Fig. S8H). Taken together, these experiments and characterization suggest that KLHL6 targets the BCR and disruptions in the gene result in an aberrant BCR phenotype.

### Malfunctions of KLHL6 Mutants Extend Beyond Proximal BCR Signaling Homeostasis

Finally, to address these strikingly different phenotypes that the different KLHL6 mutants induced to lymphoma cells, we sought to investigate their molecular characteristics beyond the BCR components.

**Figure 7.**
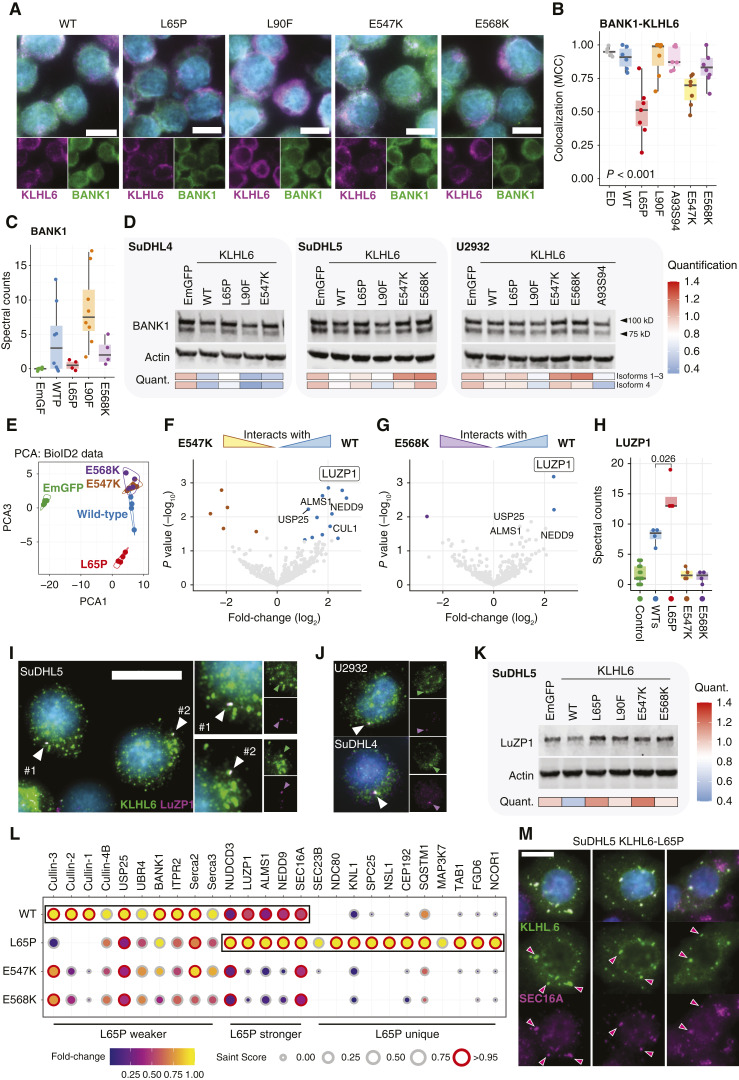
Recurrent KLHL6 mutations extend their pathogenic malfunctions to BANK1 and LuZP1. **A,** Representative IF microscopy images of SuDHL4 cells transduced with select KLHL6 constructs immunostained for endogenous BANK1, KLHL6, and DNA (Hoechst). WT overexpression; Scale bar, 10 µm. **B,** BANK1-KLHL6 colocalization for different KLHL6 overexpression constructs in SuDHL4 cells measured from fluorescent microscopy images. Staining from endogenous KLHL6 (ED) from the nontransduced parental cell line used as a control. Each point represents the correlation coefficient of one analyzed image. *P* value calculated with the Kruskal–Wallis test. MCC, Manders’ correlation coefficient. **C,** Spectral counts of BANK1 peptides from different affinity-purified in SuDHL5 pull-downs according to WT and mutant Strep-tagged baits. Dots represent biological pull-down replicates. **D,** Western blot analyses of BANK1 in SuDHL4, SuDHL5, and U2932 cell lines transduced with EmGFP control and nontagged KLHL6 WT and mutant constructs. Quantification of the signal intensity was normalized to a housekeeping control (β-actin) and compared with signal from EmGFP transduced cells. **E,** PCA of mass spectrometry data from SuDHL5 cells transduced with different BioID2-tagged constructs. **F** and **G,** Volcano plots of differentially interacting proteins between WT and Kelch mutant (E547K and E568K) BioID2-tagged KLHL6 baits. **H,** Spectral counts of LuZP1 in BioID2 interactome screens from SuDHL5 cells transduced with the appropriate constructs. Controls include in-house purifications with various unrelated BioID2 purifications from SuDHL5 cells to demonstrate the level of background noise for LuZP1 prey. Mann–Whitney *U* test. *P* value for comparison between WT and KLHL6^L65P^ counts is shown. **I** and **J,** Representative IF images showing colocalization between endogenous KLHL6 and centrosomal LuZP1 proteins in unmanipulated lymphoma cell lines. Arrowheads point LuZP1 signal per representative cell. Scale bar, 20 µm. **K,** Western blot analysis of cell lysates from SuDHL5 cells transduced with KLHL6 WT and mutant constructs or EmGFP control. **L,** Balloon plot of SAINT analysis of interactome screening with BioID2-tagged KLHL6 baits. Size of the balloons represents SAINT score with high-confidence interactions (≥0.95) annotated with red borders. Fold-change of spectral counts represented with color fill. **M,** Representative IF images of SuDHL5 cells overexpressing *KLHL6*^L65P^ mutant stained for KLHL6 and SEC16A. Red arrowheads indicate co-staining vesicles. Scale bar, 10 µm.

In DLBCL samples, *BANK1* gene expression was linked to opposing molecular features to KLHL6 expression, including higher expression associated with ABC subtype, mutations in BANK1 interactor *MYD88* (47) and association with S3 and S4 B-cell states (Supplementary Fig. S9A–E). We introduced KLHL6 constructs to SuDHL4 cells, with KLHL6^GC+^ phenotype and high BANK1 expression, and observed the strongest colocalization between BANK1 and KLHL6^L90F^ and KLHL6^A93D-S94C^ mutants in the cytosol (Fig. 7A and B). By contrast, colocalization was compromised between BANK1 and KLHL6^L65P^ and Kelch domain mutants as supported by AP-MS interactome data with the strongest association between BANK1 and KLHL6^L90F^ (SaintScore > 0.95; Fig. 7B and C; Supplementary Table S5A). KLHL6^L90F^ and KLHL6^A93D-S94C^ promoted BANK1 degradation even more effectively than WT KLHL6 overexpression across all the tested cell lines, whereas KLHL6^L65P^ and the Kelch domain mutations failed to promote BANK1 downregulation (Fig. 7D). These findings together with our AP-MS data supporting homodimeric CRL-formation highlight that KLHL6^L90F^ remains functional for the downregulation of BANK1, whereas the other recurrent BTB domain mutant KLHL6^L65P^ exhibits loss of function.

To gain additional insights into the pathogenic roles of Kelch domain mutants, we performed PL-MS with mutant constructs in SuDHL5 cells. According to the principal component analysis (PCA), the proximity interactors of the Kelch domain mutants KLHL6^E547K^ and KLHL6^E568K^ overlapped and differed from those of the WT bait (Fig. 7E). Explaining this difference, the KLHL6 interactor LuZP1 was the most prominently lost interaction partner by both Kelch domain variants in the high-throughput data (Fig. 7F and G; Supplementary Table S5B and S5C). By contrast, the pull-downs with KLHL6^L65P^ bait contained significantly more LuZP1 peptides, indicating substrate trapping properties of this poorly Cullin-3–associated bait (Fig. 7H). Co-staining of LuZP1 and KLHL6 in DLBCL cells revealed that one cytosolic KLHL6 punctum colocalized with centrosomal LuZP1 protein by IF microscopy (Fig. 7I and J). Remarkably, only WT KLHL6 overexpression promoted LuZP1 downregulation, whereas recurrent BTB and Kelch domain KLHL6 mutants did not (Fig. 7K).

Finally, we explored the BioID2 interactions of *KLHL6*^L65P^ bait (Supplementary Table S5D). Proximity labeling confirmed the poor association between *KLHL6*^L65P^ and Cullin-3 and other Cullin family scaffolds, USP25 and UBR4 (Fig. 7L). However, *KLHL6*^L65P^ gained unique interactions and was more strongly associated with KLHL6 interactor SEC16A that colocalized with aberrant KLHL6^L65P^ vesicles in SuDHL5 cells (Fig. 7L and M). Although mostly descriptive, the above data reveal both shared and unique substrates between recurrent KLHL6 variants that extend beyond dysregulation of BCR expression and signaling.

## Discussion

The landscape of recurrently mutated protein-coding genes in DLBCL is well established (25, 26, 31, 48), but their functional implications have remained largely uncharacterized. Through translational investigation including comprehensive proteomic analyses of KLHL6 and its recurrent mutants in the malignant B-cell context, we discovered novel functionalities for this protein. Our study revealed that BTB domain mutants of KLHL6 fail to heterodimerize and promote degradation of their substrates CD79A, CD79B, and IgM, which constitute the BCR complex. Expression of the BTB-mutant *KLHL6*^L65P^ increased BCR surface levels on the plasma membrane and induced a hyper-responsive phenotype to BCR signaling. Moreover, additional functional anomalies exhibited by recurrent BTB and Kelch domain mutants induced phenotypic changes that distinguish them from total genomic ablation of *KLHL6*. Together, our findings reshape the pathogenic role of *KLHL6* mutations in lymphoma, which have previously been postulated as passenger mutations arising from aberrant somatic hypermutation or resulting in complete loss of function (24, 33). We anticipate our findings to extend to other B-cell diseases with *KLHL6* mutations, such as CLL and B-cell autoimmunity besides B-NHL.

Discovery of new pathogenic BCR signaling mechanisms could aid rational stratification of patients for different therapeutic interventions (49). The absence of the KLHL6^GC+^ phenotype in almost half of ABC DLBCLs could be permissive to the oncogenic chronic active mode of BCR signaling in which sustained proximal BCR signaling and evasion of anergy through increased plasma membrane density of self-reactive BCRs represents a hallmark. Previous studies have demonstrated that lymphoma cells can unlock this oncogenic strategy through CD79B/A mutations, which result in increased BCR surface levels through hindered BCR internalization and loss of negative regulation by phosphorylation by LYN (10). We showed that *KLHL6* loss and recurrent BTB mutations add to this hallmark by increasing protein and membrane levels of BCR signaling subunits in cell lines and patients. These phenotypic characteristics have been shown to impact responses to small-molecular inhibitors (50) and could render these lymphomas sensitive to the anti-CD79B antibody–drug conjugate polatuzumab vedotin with promising efficacy especially in the ABC DLBCLs (51, 52). Furthermore, association and potential cooperation between loss of KLHL6^GC+^ and *MYD88* mutations could promote synergistic BCR and Toll-like receptor 9 (TLR9) signaling via oncogenic My-T-BCR complex, which is targetable with small-molecule inhibitor ibrutinib (18, 53, 54). Examining KLHL6 in the tumor tissues of patients treated with these regimens could reveal a biomarker role for *KLHL6* mutations and KLHL6^GC+^ phenotype to predict responses besides being a prognostic marker in the ABC DLBCL.

Recurrent BTB domain mutations and the related loss of KLHL6^GC+^ phenotype could add to chronic active mode of BCR signaling by disrupting surface BCR homeostasis. Most established CRL complexes, such as KEAP1-CRL, rely on BTB domain mediated homodimer formation and their accurate quality control and spatiotemporal regulation is the basis of their proper function (41, 55). Intriguingly, we observed specific disruption of heterodimers whereas homodimerization remained unaffected. These patterns could be related to KLHL6 regulation and could give rise to dominant negative features of especially the *KLHL6*^L65P^ mutant with poor binding to Cullin-3. However, other phenotypic changes and the opposing impact of Kelch domain mutations warrant further explanations. Some aberrant characteristics of KLHL6 mutants might not relate to perturbed BCR surface levels and could arise from disruptions relating to other KLHL6 interactors, such as BANK1 and LuZP1, or they could be linked to previously described mRNA dysregulation and activation of NF-κB signaling through Roquin-2 (33). Our findings provide foundations for future biochemical investigations relating to the regulation of KLHL6-CRLs E3 ligase function that could expose completely new therapeutic strategies to KLHL6 dysregulated diseases.

Multiple mutations in different genes contribute to lymphoma growth by disrupting surface BCR homeostasis. Here, we linked mutational dysregulation of KLHL6 as a mechanism to oncogenic BCR signaling. Distinct impacts of different KLHL6 variants on BCR plasma membrane levels and signaling raise questions for future studies to determine what are the BCRs coupled with these different mutations and what antigens, whether self or foreign, they can recognize. We expect our findings will unlock the exploration of therapeutic BCR targeting following *KLHL6* dysregulation in B-cell neoplasia and autoimmunity.

## Methods

### Human Subjects

The study was approved by the Ethics Committee of Helsinki University Hospital (HUH), Finland; the National Authority for Medicolegal Affairs, Finland; and Institutional Review Board. The patients treated in Nordic phase II trial protocols signed an informed written consent before study enrollment and sample collection was performed in accordance with the Declaration of Helsinki.

#### Tissue Microarrays and Whole-Tissue Sections

We utilized previously prepared tissue microarrays (TMA) constructed from residual formalin-fixed, paraffin-embedded (FFPE) tumor tissue from diagnostic DLBCL biopsies treated in the Department of Oncology, HUH (56). One to four cores were punched from representative regions of diagnostic tumor tissues. The patients with evaluable TMA cores that had available molecular data produced previously (34) comprised the discovery cohort of the study. Additionally, whole-tissue FFPE sections from available tissues of patients with DLBCL treated according to Nordic dose-densified chemoimmunotherapy protocols were utilized in the training set of tumors to optimize staining procedure, characterize KLHL6 staining prior to discovery set analysis, and as an expansion series to characterize KLHL6 mutants on the protein level by IF and IHC microscopy. These samples were residual tissues from diagnostic biopsies of young (<65 years of age) clinically high-risk (age adjusted IPI ≥ 2) patients with primary DLBCL. Molecular subtype by Hans’ algorithm is annotated in the representative figures.

#### Mutational and Gene Expression Data Analysis

Mutational data for the discovery set was available from the original publication and the RNA sequencing gene expression data of log_2_-transformed fragments per kilobase of transcript per million mapped reads (FPKM) per gene was obtained from the corresponding authors [European Genome-Phenome Archive: EGAD00001003600; ref. 34]. Variant calls from a recent re-analysis of the discovery cohort exomes were available and prioritized in the study (bioRxiv 2023.11.21.567983) and complemented with one in-house validated *KLHL6* L65P mutation (Supplementary Fig. S8G). LymphGen classifications (30) were determined using the online tool with revised mutations, without considering copy number alterations and using *BCL2* and *BCL6* translocations determined previously for available cases (56). Additional DLBCLs with BTB domain mutations were screened with capillary sequencing of *KLHL6* exon one from fresh-frozen tissue-derived DNA available from expansion set of tumors and one Kelch domain mutation was detected from targeted deep sequencing data (Supplementary Fig. S8H). Meta-analysis of *KLHL6* mutations was performed by pooling together *KLHL6* mutations as reported or obtained from corresponding authors in the publications listed in Supplementary Table S2A (31, 34, 38, 48, 57–62).

Uniform manifold approximation and projection analysis (UMAP) of 624 DLBCL transcriptomes (34) was performed using R package UMAP (version 0.2.10.0). B-cell ecotypes and cell states were obtained from supplementary information of Steen and colleagues (35). Single-cell RNA sequencing–based results of *KLHL6* expression in various GC B-cell populations was characterized from supplementary files of Holmes and colleagues (63).

### Genetic Manipulation of Lymphoma Cells

#### Cell Lines

SuDHL4, SuDHL5, SuDHL8, dB, HBL1, Ri-1 (Riva), U2932, NuDUL-1, Ocily3, Ocily7, Ocily8, and Ocily19 were obtained from Professor Karen Dybkaer (Aalborg University Hospital, Denmark). SuDHL10 cell line was obtained from Dr. Caroline Heckman [Institute for Molecular Medicine Finland (FIMM), Helsinki Institute of Life Science (HiLIFE), University of Helsinki, Finland]. The cells were cultured at 37°C in 5% carbon dioxide and maintained in humid conditions in their respective culture mediums as listed in Supplementary Table S6. Cell line authentication was performed for select cell lines at the Institute of Molecular Medicine Finland (FIMM, HiLIFE Unit, University of Helsinki) using the GenePrint25 system (Promega). The authentication of HBL1 with no publicly available reference genotype profile available was performed by confirming that cell line did not match with any of the available cell line profiles. The cells were confirmed negative for mycoplasma using the MycoAlert kit (Lonza).

#### Transfection

Human embryonic kidney cells (293FT) were cultured in DMEM 10% (Supplementary Table S6) and plated the day prior transfection to Petri dishes (500,000 or 1,500,000 cells to dishes 5 or 10 cm in diameter, respectively) in antibiotic-free culture medium. For 10-cm plates, the lipid-based transfection reaction was set by first adding 36 μL DharmaFECT Duo (Dharmacon) transfection agent to 1,200 μL of room temperature (RT) Opti-MEM (Gibco) medium, and the mixture was incubated for 5 minutes. Then, the 6 to 12 µg plasmids were added mixing the reaction thoroughly, and micelles were allowed to form for 20 minutes in RT prior adding the mixture dropwise to cells. After 18 to 24 hours of incubation in cell incubator, the culture medium was removed and replenished with antibiotic-containing culture medium.

#### Lentivirus Production

We used 293FT cells to produce lentiviruses for transduction. 293FT cells were kept from reaching confluence prior to virus production and plated on culture dishes with antibiotic-free medium on the day before transfection. After 24 hours, the cells were co-transfected with 2.25 to 4.5 µg lentiviral packaging plasmids (both gifts from Didier Trono, pCMVR8.74, Addgene #22036, or psPAX2, Addgene #12260), 0.75 to 1.5 µg envelope plasmids [a gift from Bob Weinberg, pCMV-VSV-G, Addgene #8454 or a gift from Didier Trono, pMD2.G (VSV-g), Addgene #12259], and 3 to 6 µg desired constructs, with the protocol described above. After an overnight incubation, the supernatant was removed and replenished with fresh antibiotic-containing culture medium. After 48 hours (72 hours from transfection), the lentivirus-containing supernatant was harvested with a syringe and run through a 0.45 μm filter. If not immediately used for transduction, the viral supernatant was aliquoted and stored in −70°C for later use.

#### Transduction

Lymphoma cell lines were transduced with the spinoculation method. The target cells, passaged the day prior to transduction, were collected 50,000 to 250,000 cells per transduction, centrifuged and the supernatant was removed. Then, the cells were suspended to 500 to 1,000 μL of lentiviral supernatant supplemented with 8 μg/mL polybrene. Spinoculation was performed by centrifugating the cells in 24-well plates or conical tubes for 2 hours with 800 *g* in RT. After spinoculation, the viral supernatant was removed, and the cells were suspended and plated in fresh culture medium. Fourty-eight hours after transduction, the cells transduced with vectors containing antibiotic selection markers were supplemented with the respective antibiotics [puromycin (0.5 µg/mL)] for positive selection of transduced cells. Cells with green fluorescent protein (GFP) as selection marker were selected with fluorescent activated cell sorting (FACS).

#### Production of Expression Constructs

The full-length human *KLHL6* cDNA was cloned from cDNA synthetized from RNA extracted from SuDHL4 cell line with available sequencing data (Cancer Cell Line Encyclopedia) and in-house RNA sequencing data indicating abundant transcript expression encoding a WT protein (Uniprot ID Q8WZ60, 621 amino acids). Constructs with C-terminal Strep-HA and BioID2 tags were manufactured by restriction-ligation cloning and followed by their transfer into mammalian overexpression vector. Full-length coding sequence of KLHL6 with and without a stop codon was PCR amplified and cloned into a C-terminal Strep-tag-HA cassette in pcDNA3.1 backbone using KpnI and EcoRV restriction enzymes to produce nontagged KLHL6 and KLHL6-strHA cDNAs, respectively. Constructs of KLHL6 fused to C-terminal BioID2 were cloned into MCS-BioID2 backbone with restriction-ligation cloning with BspEI. For gateway cloning into lentiviral mammalian expression vector pLex_307 (a gift from David Root, Addgene #41392), the complete CDS of each construct was PCR amplified with gateway recombination compatible primers for recombination into entry vector (pENTR-223). Purified PCR products were subsequently destination cloned by L.R reaction (Gateway Cloning, Life Technologies, Thermo Fisher Scientific) into pLex_307. Additionally, the C-terminus V5-tagged constructs of KLHL7, KLHL26 and BANK1 were prepared from ORFeome collection of pENTR221 gateway compatible cDNAs by L.R reaction into destination vector pLex_307. The clones without stop-codons for *BANK1* (clone id: 100008707), *KLHL7* (100068501) and *KLHL26* (100073736) were used. The gateway cloning was performed at the Genome Biology Unit supported by HiLIFE and the Faculty of Medicine, University of Helsinki, and Biocenter Finland.

#### Site-Directed Mutagenesis

Site-directed mutagenesis of tagged and nontagged KLHL6 constructs was performed for pertinent pENTR-223 entry vectors using QuikChange II Site-directed mutagenesis kit (Agilent Technologies, Santa Clara, CA, USA) according to the manufacturer’s protocol using half the reaction volumes. Primer sequences for mutagenesis were designed using the manufacturer’s Primer Design Program online tool (Supplementary Table S6). After the reactions, the resulting plasmids were Sanger sequenced for successful mutagenesis prior to their recombination into the destination vector pLex_307.

#### CRISPR-Cas9-Mediated Gene Silencing

We designed single-guide RNA (sgRNA) constructs targeting *KLHL6* and used nontargeting sgRNAs derived from the GeCKO V2 library (identifiers HGLibA_14895 and HGLibA_14096) as controls in CRISPR-Cas9 experiments to produce isogenic knockout cell line. Recombinant DNA encoding *KLHL6* or non-mammalian sgRNA targets were cloned into lentiCRISPRv2GFP backbone [a gift from David Feldser (64), Addgene #82416] and the lentivirus particles were produced as described above. After transduction, SuDHL5 cells were cultured for multiple passages until the GFP positive cells were batch and single-cell sorted into U-bottom 96-well plates with 100 µL culture medium using Sony SH800 cell sorter. The resulting sorted batch was used to assess editing and knockout efficiency, and the appropriate number of single-cell clones were expanded and screened for isogenic frameshift mutations leading to premature stop codons with capillary sequencing.

Comparison between *KLHL6* and *KLHL7* silencing was performed in SuDHL4 cells using another set of sgRNAs (Supplementary Table S6). SuDHL4 cells were transduced with lentiCas9-EGFP (a gift from Phil Sharp and Feng Zhang, Addgene plasmid #63592) and an isogenic GFP+ single cell line was cloned as described above using the SH800 sorter. Then, two *KLHL7* targeting guides from the Brunello library, two targeting *KLHL6* and two control guides targeting intergenic regions of chromosome 2 and *MYC* were cloned to lentiGuide-Puro (a gift from Feng Zhang; Addgene plasmid #52963) vector, transduced to Cas9-EGFP+ SuDHL4 cell line and positively selected using puromycin. Gene-silencing efficacy of *KLHL7* targeting guides was evaluated with capillary sequencing of target regions with Inference of CRISRP Editing (ICE) software (Synthego) indicating knockout scores of 71 and 75 for KLHL7.3 and KLHL7.4 sgRNAs, respectively. Finally, isogenic clones of edited cells were single cell cloned as described above, screened for frameshifts leading to premature stop codons with capillary sequencing and analyzed for their BCR phenotypes (Supplementary Fig. S5G).

#### RNA Interference–Mediated Knockdown

Short hairpin RNAs (shRNAs) were used to knockdown KLHL6 expression in lymphoma cell lines. Bacterial clones with plasmids (pLKO.1) encoding *KLHL6* targeting shRNA library (TRCN0000159143, TRCN0000159320, TRCN0000161116, TRCN0000161652, TRCN0000162118, TRCN0000164063) by the RNAi Consortium (TRC) were from Sigma-Aldrich MISSION shRNA library, distributed by Genome Biology Unit core facility. Knockdown efficiency was evaluated by qPCR and Western blotting, and shRNAs TRCN0000159143 and TRCN0000162118 referred in the manuscript as “sh_#9143” and “sh_#2118,” respectively, were selected and used in the representative knockdown experiments. Non-mammalian targeting scrambled (SHC002) and GFP targeting (SHC003) control shRNA plasmids (pLKO.1) obtained from the laboratory of Jorma Keski-Oja were used as RNAi controls.

### Capillary Sequencing

Capillary sequencing for PCR amplified select tumor DNA and cellular DNA or plasmid DNA was performed at the Institute of Molecular Medicine Finland (University of Helsinki supported by HiLIFE and Biocenter Finland) using ABI3730XL DNA Analyzer (Applied Biosystems) and BigDye version 3.1 (Applied Biosystems) and the data were manually examined using the SnapGene Viewer tool (version 4.2.6).

### Cell Viability Measurements

Cell viability and growth was measured using CellTiter-Glo 2.0 Cell Viability Assay (CTG, Promega) according to the manufacturer’s protocol and the luminescence was measured with FLUOstar Omega Microplate Reader (BMG Labtech). For monitoring growth of SuDHL5 and U2932 cells transduced with different KLHL6 or control constructs, 1,000,000 live cells were plated in 10 mL of cell culture medium and baseline cell viability was measured after 24 hours. Then, the viability of the cells was measured every 24 hours using CTG in three technical replicates and the cell culture was topped up to 10 mL with fresh culture medium. The culture was maintained until confluency and the measured luminescence was normalized to the signal of the first measurement.

#### Viability after BCR Cross-linking

Lymphoma cells were cultured in their respective culture medium and passaged the day before the experiments. On the day of the stimulation, 25,000 cells per well were plated into white flat-bottom 96-well plates in 50 µL of their respective culture medium. Goat anti-human IgM F(ab′)2 (Southern Biotech) was first diluted in PBS and then mixed with cell culture medium to be added to the cells in 1:1 ratio for desired final concentrations (0.3, 1, 3, and 10 µg/mL). Unspecific purified goat IgG (Supplementary Table S6) at concentration 10 µg/mL was used for control cells. The viability of the cells was measured after 48 hours of stimulation using CTG. The measured luminescence was normalized to the signal of the control treatment.

### Calcium Release Assay

Fluo-8 Calcium Flux Assay Kit (Abcam, ab112129) was used according to manufacturer’s instructions to assess kinetics of BCR cross-linking induced Ca^2+^ flux in SuDHL5 cells. For the experiments, 250,000 SuDHL5 cells per well were plated on black 96-well plates coated with Poly-L-lysine and incubated in dye-loading solution for 1 hour (30 minutes in cell culture incubator and 30 minutes in RT). Then, the plates were centrifugated once, the supernatant was removed, 100 µL of Hank’s Balanced Salt Solution (HBSS, Gibco) was added and the prepared plates that were placed in FLUOstar Omega microplate reader. In the experiment, the wells were injected with 100 µL goat anti-human IgM F(ab′)2 or unspecific goat IgG (10 µg/mL) using plate reader’s injectors and the fluorescence was monitored at excitation/emission 490/525 nm per second for 5 minutes.

### Western Blot Analyses

Cells were cultured as described above and passaged the preceding day before harvesting. Proteins were extracted by lysing the cell pellets in RIPA Lysis Buffer with brief sonication. The lysate was clarified by centrifugation and the protein concentrations were determined using DC Protein Assay and measured with FLUOstar Omega. The separately processed cytoplasmic and nuclear protein fractions were extracted from 10 μL cell pellets using NE-PER Nuclear and Cytoplasmic Extraction Reagents kit (Thermo Fisher) according to the manufacturer’s protocol. All lysates were supplemented with protease and phosphatase inhibitor cocktails if phosphoproteins were investigated. Laemmli sample buffer supplemented with ß-mercaptoethanol was added to the lysates, which were then denaturated in a 95°C heat block for 5 minutes.

The samples were run in 4% to 15% precast gradient gels (Bio-Rad). After electrophoresis (100V, 75–80 minutes) in electrophoresis chambers, the proteins were transferred to a 0.2 µm nitrocellulose membrane with a Trans-Blot Turbo Transfer system (3–7 minutes, 2.5 A, up to 25 V). Total protein load was detected with No-Stain Protein Labeling Reagent (Invitrogen) for normalization. The membrane was blocked and the indicated primary and secondary antibodies were diluted in 5% milk in TBS-Tween (0.1% Tween 20). The detection of proteins was performed using Odyssey Fc Imaging System and Image Studio Lite software (LI-COR Biosciences; Supplementary Table S6) or Azure 500 Imaging System (Azure Biosystems; Supplementary Table S6). Quantification of the protein levels and adjustment of images for publication were performed using the standard tools of AzureSpot Pro or Image Studio Lite software with default settings (Supplementary Table S6). All unprocessed Western blot images of the figures are shown in Supplementary file Unprocessed Western blots, and raw image files and additional blots can be inquired from the corresponding authors.

### qPCR

qPCR to determine KLHL6 mRNA transcript levels in cell lines and genetically manipulated lymphoma cells. RNA was isolated with Nucleospin RNA kit (Macherey–Nagel) according to manufacturer’s protocol and the complementary DNA (cDNA) was synthetized from 500 ng of RNA using iScript cDNA synthesis kit (Bio-Rad). The cDNA was prepared according to the manufacturer’s protocol and diluted ½ with ddH_2_O. The qPCR reactions were prepared on 96-well plates in triplicate using 5 μL of TaqMan Universal PCR Master Mix and 0.5 μL of TaqMan probes (Applied Biosystems), 2.5 μL of molecular grade H_2_O and 2 μL of diluted cDNA and run with ABI 7500 Fast Real-time PCR (Thermo Fisher) equipment. The TaqMan probe assays (Thermo Fisher) used for *KLHL6* (Hs01092201_m1) were normalized to reference housekeeping assay for *GAPDH* (Assay ID Hs02786624_g1). The data was analyzed and the relative quantifications were calculated using 7,500 Fast Real-Time PCR System Software (Applied Biosystems, version 2.3).

### IHC

IHC staining was performed using 4- to 5-μm-thick tissue and TMA FFPE sections attached on microscopy slides. After deparaffinization with xylene, the sections were permeabilized using Tris-EDTA buffer (10 mmol/L Tris base, 1 mmol/L EDTA solution, 0.05% Tween 20, pH 9.0) and blocked with hydrogen peroxide. Primary antibodies were diluted in Normal antibody Diluent (ImmunoLogic) and incubated overnight in +4°C. Unconcentrated mouse anti-human KLHL6 antibody (clone 92C, CNIO, Supplementary Table S6) was used at 1:3 dilution (65). After primary antibody incubation, the sections were treated with BrightVision anti-mouse HRP (ImmunoLogic), ImmPACT Diaminobenzidine Peroxidase Substrate Kit (Vector), and stained with Mayer’s Hematoxylin Solution (Sigma-Aldrich). The slides were washed two times with PBS for 5 minutes between all the steps, and finally for 10 minutes under running water after hematoxylin treatment.

IHC was analyzed together with an expert hematopathologist using a dual-head microscope. Light microscopy figures for publication were obtained from stained tissue sections scanned with Pannoramic 250 Flash III, with 20× air objectives (3DHISTECH, Budapest, Hungary) and analyzed in Pannoramic viewer environment (version 1.15.4).

### Immunofluorescence Microscopy

#### Preparation of Cell Lines

Lymphoma cells were passaged the day before their harvest for IF analysis. Briefly, the cells growing in their respective standard culturing conditions were collected and washed once with ice-cold PBS. Then the cells were fixed with 4% paraformaldehyde in PBS gently rocking on ice for 15 minutes. Then the cells were washed two times with PBS and H_2_O prior to their attachment on microscopy slides. Round areas (∼1 cm ∅) for fixed lymphoma cells were encircled with hydrophobic marker and the microscopy slides were coated with poly-L-lysine (Sigma) by incubating for 5 minutes. Desired numbers of cells (typically 200,000) suspended in PBS were plated on coated regions and were allowed to sediment, attach, and the glasses were allowed to dry o/n. The attached cells were washed with PBS and then permeabilized with 0.1% Triton X-100 in PBS by incubation of 5 minutes and washed three times with PBS. Then, the cells were blocked with 3% bovine serum albumin (BSA) in PBS, washed once and stained with primary antibodies diluted in 3% BSA-PBS. After 1 hour of incubation in RT, the cells were washed three times with PBS and incubated for 30 minutes protected from light in RT with secondary antibodies in 3% BSA-PBS. After one wash with PBS, the DNA was stained with Hoechst (2 µg/mL) for 5 minutes. Then the glasses were washed twice with PBS and the slides were dipped in ddH_2_O and allowed to dry briefly. Finally, the cells were mounted with ProLong Gold (Invitrogen) and coverslips, and the glasses were allowed to dry before IF microscopy analysis.

#### Preparation of Tissue Sections

IF analysis of KLHL6 and select markers in human tissues was performed using two or three fluorochromes with a modified staining protocol described previously (66). Tissue and TMA sections (4–5 μm thick) were prepared, deparaffinized and blocked as described above in the IHC section. After endogenous peroxidase blocking, the slides were blocked with 15 minutes incubation in 10% normal goat serum (NGS, Gibco) TBS-T. Then, the 100 μL of diluted primary antibody (mouse anti-human KLHL6) in 10% NGS TBS-T was added and the slides were incubated in +4°C humid conditions o/n. The slides were then washed three times with TBS-T (5 minutes) and 100 μL of secondary antibody (goat anti–mouse HRP, Brightvision) was added. The slides were incubated 30 minutes and washed three times with TBS-T. Then 100 μL of tyramide conjugated with Alexa Fluor 488 was added according to manufacturer’s instructions (Thermo) and incubated for 15 minutes RT until the reagent was washed away with three TBS-T washes and once with H_2_O. After the tyramide signal amplification reaction, the first primary antibody was denatured by repeating the antigen-retrieval step. Then the slides were washed once with H_2_O and once with TBS-T and blocked again with 10% NGS TBS-T. Next set of primary antibodies was added in 10% NGS TBS-T and incubated o/n. The next morning, the slides were washed three times with TBS-T and the secondary antibodies (diluted 1:300, AF647 and AF750) and 4′,6-diamidino-2-phenylindole2-phenylindole (DAPI; at 1.7 μg/mL concentration) in TBS-T were incubated on the slides for 45 minutes in RT. After three washes with TBS-T and once with H_2_O, the slides were allowed to dry 1.5 hours in +37°C until mounting with ProLong Gold reagent and placing coverslips. Antibodies used for IF analyzes and their concentrations in parenthesis were as follows: mouse anti-human KLHL6 (CNIO, 1:3), rabbit anti-human CD79B (1:250), rabbit anti-human GM130 (1:3,000 for lymphoma cell lines, and 1:500 for FFPE tissues), rat anti-human Ki-67 (1:150), rabbit anti-human CD23 (1:100), rabbit anti-human SEC16A (1:100).

#### Colocalization Analysis

Colocalization analyses were performed with CellProfiler (version 3.1.8). Pixel-based colocalization analysis was conducted for the whole images and six to seven images were analyzed per construct. All the thresholds were determined using Otsu two-class thresholding method, and the images were masked accordingly to exclude extracellular spaces and the nuclei of the cells. Mander’s colocalization coefficients were used as a measure of colocalization.

#### IF Microscopy and Scanning

The slides were imaged using a Zeiss Axio Imager upright epifluorescence microscope with Zen 2.3 lite acquisition software. Optical sectioning microscopy was performed using the same microscope equipped with ApoTome (Zeiss). The objectives used were EC Plan-Neofluar 40×/1.30 Oil, Plan Apochromat 63×/1.40 Oil, and EC Plan-Neofluar 100×/1.30 Oil. The samples were imaged using HXP 120 V fluorescent light source and acquired using Hamamatsu Orca Flash 4.0 LT B&W camera. The imaging was performed at the Biomedicum Imaging Unit, University of Helsinki, with the support of Biocenter Finland.

IF scanning of the TMAs and select tissue sections was performed with Zeiss Axio Scan.Z1 (Zeiss, Germany) equipped with EC Plan-Neofluar 20× objective (NA 0.8), Colibri seven light source and 112 HE LED filter set. Quantification of CD79B signal was performed in Zen 2.6 lite software (Zeiss). Briefly, representative TMA punches were quantified for fluorescence in representative regions of tumor cells for AF647 signal with Zen 3.1 lite software.

### Analysis of Protein Interactomes

#### Affinity and Proximity Protein Purification

Interaction screens with AP with Strep-tag and proximity labeling (PL) with BioID2-tag were based on at least four biological replicates with 1 to 1.5 × 10^9^ cells per pull-down. The cells stably expressing the baits were cultured in their standard conditions into large volume, washed and the resulting pellet was snap-frozen with liquid nitrogen. Cells pellets prepared for biotinylation-based PL with BioID2-tag experiments were cultured with no additional biotin supplementation as RPMI contained biotin (0.2 mg/L) and no significant increase in protein biotinylation was observed with biotin supplementation.

For AP with Strep-tag, the cell pellets were thawed and lysed in 3 mL of ice-cold lysis buffer [0.5% IGEPAL, 50 mmol/L HEPES (pH 8.0), 150 mmol/L NaCl, 50 mmol/L NaF, 1.5 mmol/L NaVO3, 5 mmol/L EDTA, with 0.5 mmol/L PMSF and protease inhibitors (Sigma-Aldrich). For PL protein purification approach with BioID2-fused baits, the cell pellets were thawed in 3 mL of ice-cold lysis buffer [0.5% IGEPAL, 50 mmol/L HEPES (pH 8.0), 150 mmol/L NaCl, 50 mmol/L NaF, 1.5 mmol/L NaVO3, 5 mmol/L EDTA, 0.1% SDS, 0.5 mmol/L PMSF, and protease inhibitors]. Proximity lysates were sonicated before adding Benzonase Nuclease (Santa Cruz Biotechnology, sc-202391). Cleared lysate was obtained by centrifugation (16,000 *g* for 20 minutes), and the lysate was subjected to a one-step purification using Strep-Tactin Sepharose resin (IBA). The purified protein complexes were reduced, alkylated, and digested to peptides for MS analysis. The peptides samples were desalted with C18 macrospin columns (Nest Group) and resuspended for mass spectrometry analysis. A detailed description of the method used here can be found in a previous protocol (67).

#### Liquid Chromatography–Mass Spectrometry

The study was carried out using a Q Exactive Hybrid Quadrupole-Orbitrap Mass Spectrometer (Thermo Fisher) with Xcalibur version 2.0.7 SP1 (Thermo Fisher) linked to an EASY-nLC 1000-system through an electrospray ionization sprayer (Thermo Fisher). Four to eight biological replicates per condition were used for each sample for interaction discovery experiments and an equal amount of peptide samples was loaded in the instrumentation for each analysis. Peptides were eluted and separated with a C-18-packed pre-column and an analytical column at a flow rate of 300 nL/minutes using a 60-minute buffer gradient from 5% to 35% buffer B (buffer B: 0.1% formic acid in 98% acetonitrile and 2% HPLC-grade water; buffer A: 0.1% formic acid in 2% acetonitrile and 98% HPLC-grade water), followed by a 5-minute gradient from 35% to 80% buffer B and a 10-minute gradient from 80% to 100% buffer B. Peptides were analyzed in a data-dependent acquisition mode with a 70,000 resolution FTMS complete scan (200–2,000 m/z) and a higher energy collision dissociation scan of the top 20 most abundant ions. For protein identification, Thermo.RAW files were searched using the Sequest search engine against human entries in the UniProtKB/SwissProt database (http://www.uniprot.org/) with 15 ppm MS1 tolerance and 0.05 Da fragment mass tolerance. Carbamidomethylation of cysteine was regarded as a static alteration, whereas methionine oxidation, biotinylation of lysine, and N-termini biotinylation were defined as variable modifications. The provided findings were based on peptides assigned with high confidence in Proteome Discoverer (Thermo Fisher) and a 1% false discovery rate (FDR) by Percolator. Protein purifications and mass spectrometry were performed in the Proteomics Unit at the Institute of Biotechnology supported by University of Helsinki, HiLIFE, and Biocenter Finland.

#### Identification of the High-Confidence Interactions with SAINT and DESeq2

As a statistical strategy for identifying high-confidence interactions from AP-MS and PL-MS data, the online tool (http://proteomics.fi/) integrated with Significance Analysis of INTeractome (SAINT) express version 3.6.0 (68, 69) was utilized. Four to eight EmGFP control runs (four Strep-tagged and four nontagged for AP approach, BioID2-tagged for PL experiments) were used as control counts for each hit. High-confidence interactions (HCI) were defined by an estimated Bayesian FDR of ≤0.01. Additionally, the CRAPome database (70) with a cutoff frequency of ≥20% (≥82), except for an average spectral count fold change ≥3 was used to further flag the possible false positive hits. As a complementary approach to SAINT, we applied DESeq2 (71) that normalizes and accounts for dispersion in count data from individual purifications to determine significant differentially interacting proteins between different conditions. R package DESeq2 (version > 1.24.0) was used in the analysis. All the conclusions were evaluated with both models and manual examination of spectral counts.

##### BCR Cross-linking AP-MS Experiment

SuDHL5 cells stably expressing Strep-HA-tagged KLHL6 and EmGFP constructs were cultured into large volumes to produce ∼750 million cells per experiment. Prior to stimulation, 1.5 × 10^9^ cells were collected and sensitized for stimulation by incubation in low serum (0.5%) culture medium for 30 minutes in cell culture incubator. The sensitized cells were then collected, centrifuged, and the medium was removed. Then, the cells were suspended in 10 mL of PBS (RT), split into two 15 mL conical tubes at 1:1 ratio, centrifuged and the PBS was removed to obtain ∼ 600 μL cell slurry for parallel stimulation and non-stimulation control reactions. Then, 600 µL of goat anti-human IgM F(ab′)2 (0.5 mg/mL) or unspecific purified goat IgG unlabeled (diluted to 0.5 mg/mL with PBS) were added to cells or for stimulation and control experiments, respectively, and the reaction was incubated in RT for 60 seconds before snap-freezing in liquid nitrogen. For stimulation of control cells expressing EmGFP baits, only stimulations with anti-human IgM F(ab′)2 was performed. Western blot samples to control successful stimulation containing 10 μL of cell slurry were collected prior to and immediately following the adding of the reagent, and the control samples were snap-frozen together with the falcon tube at 60 second landmark. The samples were stored in −70°C until lysis and the AP-MS was performed in one batch according to the protocol described above.

### Flow Cytometry

#### Surface BCR Levels

The surface expression of the BCR components CD79A, CD79B, and IgG or IgM in SuDHL4, SuDHL5, OciLy7, and U2932 cells was measured by flow cytometry. The cells were stained with the following antibodies (Supplementary Table S6): anti–CD79A-PE, anti–CD79B-PerCPCY5.5, anti–CD19-APC, and anti–IgM-Pacific Blue or anti–IgG-FITC on ice for 30 minutes before analysis on a FACS Canto II (BD).

#### Signaling by Phosphorylation-Specific Flow Cytometry (Phospho-Flow)

Activation of BCR signaling was performed as previously described (50, 72). Briefly, cell lines were distributed at 5 million cells/mL and rested for 75 minutes, then stimulated with anti-IgM F(ab′)_2_ (Jackson ImmunoResearch) or anti-IgG F(ab′)_2_ (Jackson ImmunoResearch) at a final concentration of 10, 5, 2.5 µg/mL or left unstimulated for 5 minutes. Cells were fixed by adding paraformaldehyde to a 1.6% final concentration for 5 minutes at RT, then washed with PBS, and permeabilized with ice-cold methanol (final concentration > 90%) and stored in −80°C.

On the day of flow cytometry analysis, cells were rehydrated by washing twice in PBS. For barcoding, the four different activation conditions for each isogenic cell line were resuspended in PBS, and each well was incubated with a specific concentration of Pacific Blue dye (0.5, 0.125, 0.031, and 0 ng/µL) for 30 minutes in the dark. Antibody staining was performed at RT for 30 minutes bere washing and running the samples on a FACS Canto II (BD). The following antibodies from BD were used (Supplementary Table S6): anti–p-BLNK-Ax647, anti–p-BTK-Ax647, anti–p-LCK-PE, anti–p-PLC-γ-Ax488, anti–p-SYK-PE, anti–p-ERK-Ax488, anti–p-STAT5-Ax488, anti–p-p38-PE, and anti–p-AKT1-Ax647. Cytobank Software was used for data analysis, and relative phosphorylation changes were calculated using arcsinh transformation of median fluorescence intensity (MFI) relative to unstimulated cells.

### Statistical Analysis and Data

Statistical analyses were performed in R (version ≥ 3.6.1) and GraphPad Prism 9.5.1. Details about statistical testing is described in the figure legends and were nonparametric and two-sided unless otherwise indicated. *P* values < 0.05 were considered statistically significant and the significance is denoted in the figures as follows: *, *P* < 0.05; **, *P* < 0.01; ***, *P* < 0.001; and ****, *P* < 0.0001.

#### Survival Analysis

Survival analysis of the discovery cohort was performed using R packages. Survival analyses were performed with R package survival (version 2.44-1.1). Kaplan–Meier estimates were prepared and visualized with R package survminer (version 0.4.6) and cox regression models; HRs and 95% confidence intervals were visualized with forestplot R package (version 1.9).

### Data Visualization

The figures for publication were prepared in CorelDRAW 2023 (version 24.4.0.636, Corel Corporation). In general, plots were produced in R environment using ggplot2 R package (version ≥ 3.2.1). Protein–protein interaction data were imported into Cytoscape (Supplementary Table S6) for interaction network visualization. Modeling of KLHL6 3D structure by Alphafold was examined, and the images were acquired in Chimera (version 1.13.1; Supplementary Table S6). Oncoprints and lollipop plots were produced with ComplexHeatmap R package (version ≥ 2.0.0; ref. 73) and maftools R package (version 2.16.0), respectively, and finalized in CorelDraw.

### Data Availability

MS data have been deposited to MassIVE (https://massive.ucsd.edu/) with web access MSV000092728. The protein interactions from this publication have been submitted to the IMEx consortium (http://www.imexconsortium.org) through IntAct. Other data that support this study and script to reproduce the analyses are available from the corresponding author upon reasonable request.

## Supplementary Material

Table S1. Table S1 shows molecular and clinical correlates of KLHL6 protein expression.

Table S2 Table S2 shows recurrent KLHL6 mutations reported in DLBCL.

Table S3 Table S3 shows data from interactome analysis of KLHL6 in B-cell lymphoma cell lines. Strep-tag affinity purification and mass-spec peptide identification (AP-MS).

Table S4 Table S4 shows data from the analysis of protein-protein interactions of KLHL6 identified with proximity-ligation with C-terminal BioID2 constructs.

Table S5 Table S5 shows data from the co-localization analysis of KLHL6 and BANK1 and analysis of protein-protein interactions of KLHL6 variants identified with proximity-ligation with C-terminal BioID2 constructs.

Table S6 Table S6 shows a list of resources and reagents used in the study.

Supplementary Figures S1-S9 Figure S1 shows KLHL6 expression and its molecular correlates in reactive lymphoid and DLBCL tissues. Figure S2 shows recurrent KLHL6 mutations and their subcellular localization. Figure S3 shows data from affinity-purification mass-spectrometry (AP-MS) interactome analysis of Strep-tagged wild-type KLHL6 in the expansion cell line panel. Figure S4 shows data related to proximity-labeling interactome analysis of KLHL6. Figure S5 shows KLHL6 interactome upon BCR stimulation and the impact of recurrent mutations on BCR levels. Figure S6 shows the impact of KLHL6 constructs on the surface levels expression of the BCR components. Figure S7 shows the impact of KLHL6 constructs on the BCR signaling. Figure S8 shows immunofluorescent analysis and quantification of CD79B staining intensity. Figure S9 shows BANK1 expression in DLBCL.

Unprocessed Western blots Unprocessed Western blots for Figures 1E, 3I, 4E, 4F, 5A, 5S5, 5E, 5F, 5G, 5H, 7D, 7K, S5G, S5H, and S9A
