# Supplementary material for: Disruption of KLHL6 Fuels Oncogenic Antigen Receptor Signaling in B-Cell Lymphoma
Source: Blood Cancer Discov. 2024 Apr 17;5(5):331–52. doi: 10.1158/2643-3230.BCD-23-0182 (PMC11369598; doi:10.1158/2643-3230.BCD-23-0182)
Supplement: Supplementary Figures S1-S9 — Figure S1 shows KLHL6 expression and its molecular correlates in reactive lymphoid and DLBCL tissues. Figure S2 shows recurrent KLHL6 mutations and their subcellular localization. Figure S3 shows data from affinity-purification mass-spectrometry (AP-MS) interactome analysis of Strep-tagged wild-type KLHL6 in the expansion cell line panel. Figure S4 shows data related to proximity-labeling interactome analysis of KLHL6. Figure S5 shows KLHL6 interactome upon BCR stimulation and the impact of recurrent mutations on BCR levels. Figure S6 shows the impact of KLHL6 constructs on the surface levels expression of the BCR components. Figure S7 shows the impact of KLHL6 constructs on the BCR signaling. Figure S8 shows immunofluorescent analysis and quantification of CD79B staining intensity. Figure S9 shows BANK1 expression in DLBCL. [file bcd-23-0182_supplementary_figures_s1-s9_suppsf1.pdf]

Supplementary Figures and Figure Legends

Disruption of KLHL6 Fuels Oncogenic Antigen Receptor Signaling in B-cell Lymphoma

Leo Meriranta[1,2,3], Selma Sorri[1,2,3], Kanutte Huse[4,5], Xiaonan Liu[6], Ivana Spasevska[4,5], Sadia Zafar[1], Iftekhar Chowdhury[6], Olli Dufva[7], Eerika Sahlberg[1], Luka Tandarić[1], Marja-Liisa Karjalainen-Lindsberg[8], Marko Hyytiäinen[1], Markku Varjosalo[6], June H. Myklebust[4,5] and Sirpa Leppä[1,2,3]

[1] Research Programs Unit, Applied Tumor Genomics, Faculty of Medicine, University of Helsinki, Helsinki, Finland
[2] Department of Oncology, Helsinki University Hospital Comprehensive Cancer Center, Helsinki, Finland
[3] iCAN Digital Precision Cancer Medicine Flagship, Helsinki, Finland
[4] Department of Cancer Immunology, Institute for Cancer Research, Oslo University Hospital, Oslo, Norway
[5] KG Jebsen Centre for B-cell malignancies and Precision Immunotherapy Alliance, Institute of Clinical Medicine, University of Oslo, Norway
[6] Institute of Biotechnology, HiLIFE Helsinki Institute of Life Science, University of Helsinki, Helsinki, Finland
[7] Hematology Research Unit Helsinki, Helsinki University Hospital Comprehensive Cancer Center, Helsinki, Finland
[8] Department of Pathology, Helsinki University Hospital, Helsinki, Finland

Running head: Functional characterization of *KLHL6* mutations in B cells

Key words: KLHL6, B-cell lymphoma, B-cell receptor signaling, proteomics, ubiquitin

Figure S1. KLHL6 expression and its molecular correlates in reactive lymphoid and DLBCL tissues.

(A) Low-power magnification of immunofluorescent (IF) microscopy scan of a human tonsil immunostained for KLHL6 (green), Ki-67 (white), CD23 (magenta) and DNA (blue, Hoechest). White dashed box indicates germinal center show in higher resolution in Figure 1B. Images acquired with a scanner equipped with 20x objective.

(B) Low and high-power IF microscopy images of reactive human lymph node stained as in (A). Figure below shows higher resolution image of germinal center in the dashed box.

(C) Heatmap showing gene expression fold-changes per gene (rows) among different GC B cell populations (columns) reported by Holmes et al. JEM 2020. Genes with correlated fold-change to *KLHL6* were identified by the Pearson correlation ($R > 0.5$, FDR ≤ 0.1). Significantly correlated 20 genes with the highest median absolute deviation in data shown.

(D–E) Representative IF microscopy images of SuDHL5 cells stained with KLHL6 (green) and (D) Golgi-marker GM130 (magenta) or (E) endoplasmic reticulum marker Calnexin (magenta). Note the staining of endogenous KLHL6 in vesicles concentrated in the vicinity of the Golgi apparatus.

(F) Dot and box plot showing *KLHL6* gene expression (y-axis) according to KLHL6 protein expression patterns by immunohistochemistry (IHC) (x-axis) in tumor tissue samples of the discovery cohort. Mann-Whitney U test between different IHC staining types.

(G) Oncoprint of mutations in DLBCL driver genes associated with loss of germinal center-like KLHL6 IHC staining. Mutations as originally reported by Reddy et al. 2018. Fisher's exact test, *P* values corrected for multiple testing (False discovery rate, FDR). Genes mutated in ≥5% of the cases with KLHL6 IHC results available were included in the analysis.

Figure S1.

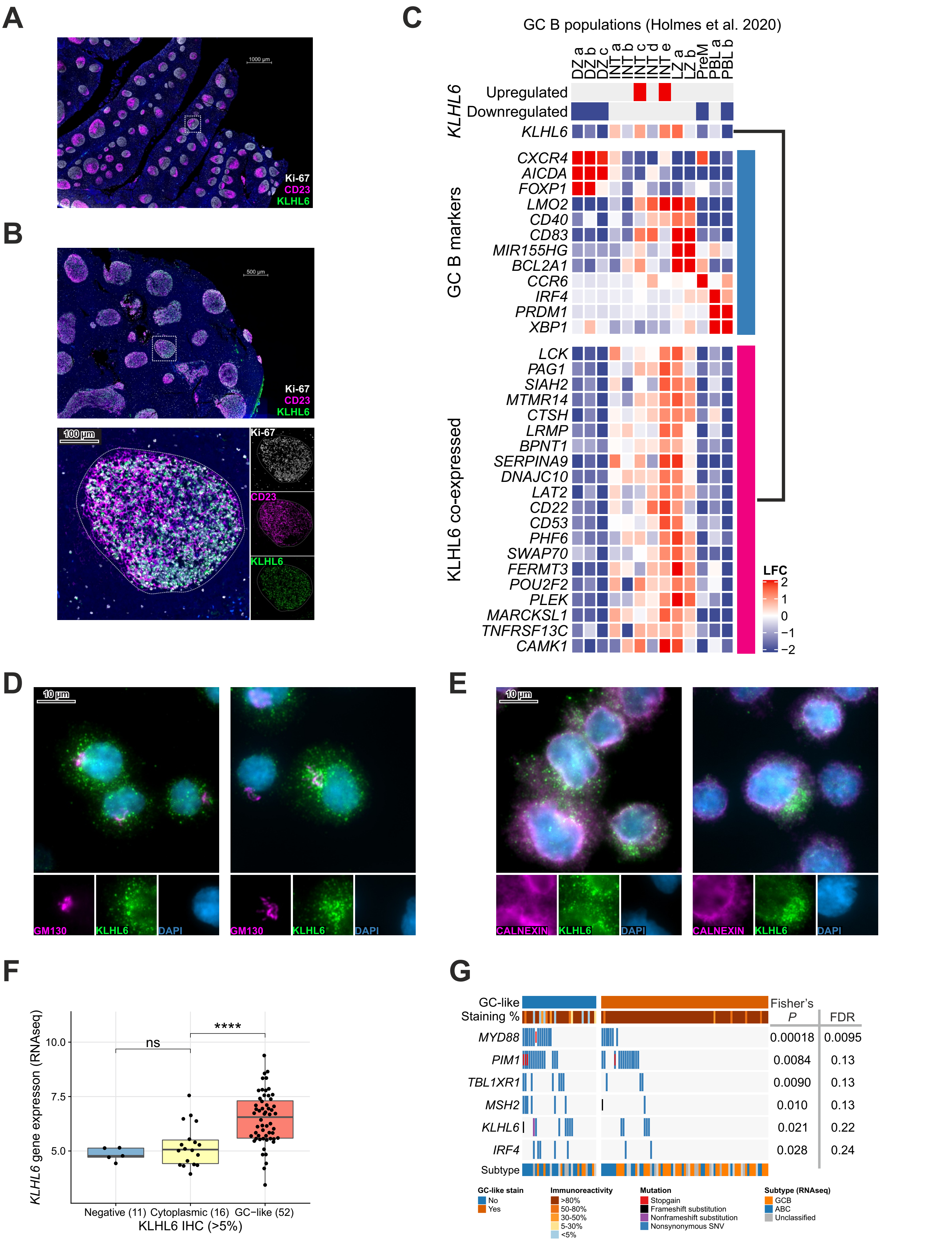
A
1000 µm
Ki-67
CD23
KLHL6
B
500 µm
Ki-67
CD23
KLHL6
100 µm
Ki-67
CD23
KLHL6
C
GC B populations (Holmes et al. 2020)
DZ a
DZ b
DZ c
INT a
INT b
INT c
INT d
INT e
LZ a
LZ b
PreM
PBL a
PBL b
KLHL6
Upregulated
Downregulated
KLHL6
GC B markers
CXCR4
AICDA
FOXP1
LMO2
CD40
CD83
MIR155HG
BCL2A1
CCR6
IRF4
PRDM1
XBP1
KLHL6 co-expressed
LCK
PAG1
SIAH2
MTMR14
CTSH
LRMP
BPNT1
SERPINA9
DNAJC10
LAT2
CD22
CD53
PHF6
SWAP70
FERMT3
POU2F2
PLEK
MARCKSL1
TNFRSF13C
CAMK1
LFC
2
1
0
−1
−2
D
10 µm
GM130
KLHL6
DAPI
GM130
KLHL6
DAPI
E
10 µm
CALNEXIN
KLHL6
DAPI
CALNEXIN
KLHL6
DAPI
F
KLHL6 gene expresson (RNAseq)
10.0
7.5
5.0
ns
****
Negative (11)
Cytoplasmic (16)
GC−like (52)
KLHL6 IHC (>5%)
G
GC-like
Staining %
MYD88
PIM1
TBL1XR1
MSH2
KLHL6
IRF4
Subtype
Fisher's P
FDR
0.00018
0.0095
0.0084
0.13
0.0090
0.13
0.010
0.13
0.021
0.22
0.028
0.24
GC-like stain
No
Yes
Immunoreactivity
>80%
50-80%
30-50%
5-30%
<5%
Mutation
Stopgain
Frameshift substitution
Nonframeshift substitution
Nonsynonymous SNV
Subtype (RNAseq)
GCB
ABC
Unclassified

Figure S2. Analysis of recurrent KLHL6 mutations and their subcellular localization.

(A) Lollipop plot of *KLHL6* mutations reported in chronic lymphocytic leukemia (n = 984) by Knisbacher et al. 2022. Y-axis, number of mutations per residue.

(B) UMAP analysis from Figure 1G with distinct KLHL6 mutations as reported by Reddy et al. 2017 highlighted.

(C) Oncoprint of *KLHL6* mutations in the discovery cohort as reported by Reddy et al. 2017 according to $KLHL6^{GC}$ IHC status distinguished for exon one and other mutations. Fisher's exact test *P* value for enrichment shown.

(D) Immunofluorescent (IF) microscopy examination of the diagnostic tumor tissue of a patient with KLHL6 L65P mutation. KLHL6 (green) and GM130 (magenta). Note the aberrant accentuation in regions apart from Golgi apparatus (red arrowhead) besides cytoplasmic expression.

(E) Representative IF images of SuDHL5 cells transduced with diverse KLHL6 mutants stained for KLHL6 (green) and GM130 (magenta).

Figure S2.

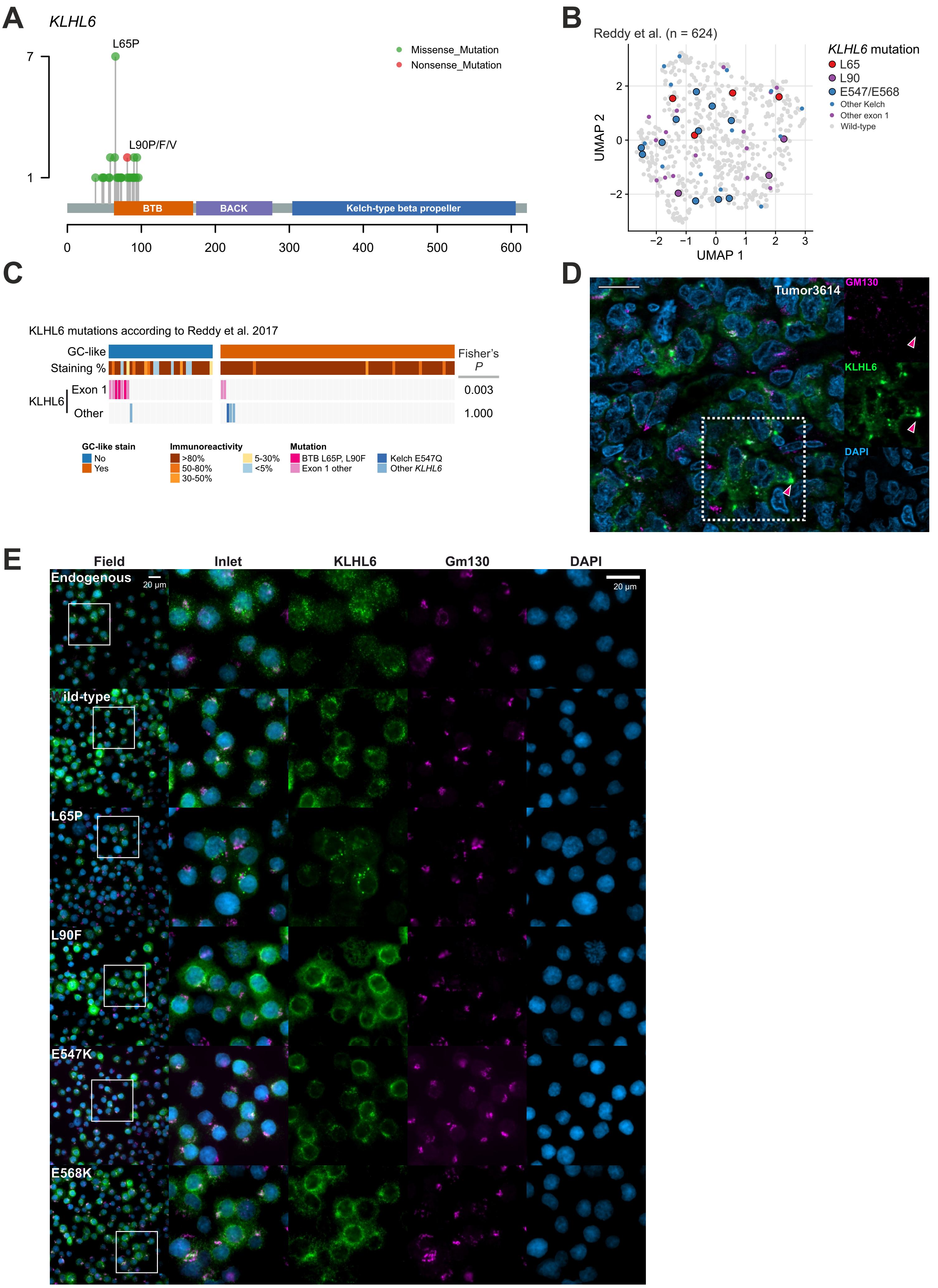

A
KLHL6
L65P
L90P/F/V
7
1
Missense_Mutation
Nonsense_Mutation
BTB
BACK
Kelch-type beta propeller
0
100
200
300
400
500
600
B
Reddy et al. (n = 624)
KLHL6 mutation
L65
L90
E547/E568
Other Kelch
Other exon 1
Wild-type
UMAP 1
UMAP 2
C
KLHL6 mutations according to Reddy et al. 2017
GC-like
Staining %
KLHL6
Exon 1
Other
Fisher's P
0.003
1.000
GC-like stain
No
Yes
Immunoreactivity
>80%
50-80%
30-50%
5-30%
<5%
Mutation
BTB L65P, L90F
Exon 1 other
Kelch E547Q
Other KLHL6
D
Tumor3614
GM130
KLHL6
DAPI
E
Field
Inlet
KLHL6
Gm130
DAPI
Endogenous
20 μm
Wild-type
L65P
L90F
E547K
E568K

Figure S3. Affinity-purification mass-spectrometry (AP-MS) interactome analysis of strep-tagged wild-type KLHL6 in the expansion cell line panel.

(A) Volcano plot of DESeq2 model for proteins interacting with KLHL6. X-axis, log2 fold-change (LFC), y-axis -log10(*P* value). Each cell line (n = 5) considered as a biological replicate and AP-MS of EmGFP constructs (one per cell line) used as controls. Prey proteins highlighted as in Figure 3B. In addition UBR4 and CD79B are annotated.

(B) Dot plot showing comparison of DESeq2 model LFCs between interactome analysis of SuDHL5 discovery cell line (x-axis) and cell line panel from A (y-axis). Color of dots as in A.

Figure S3.

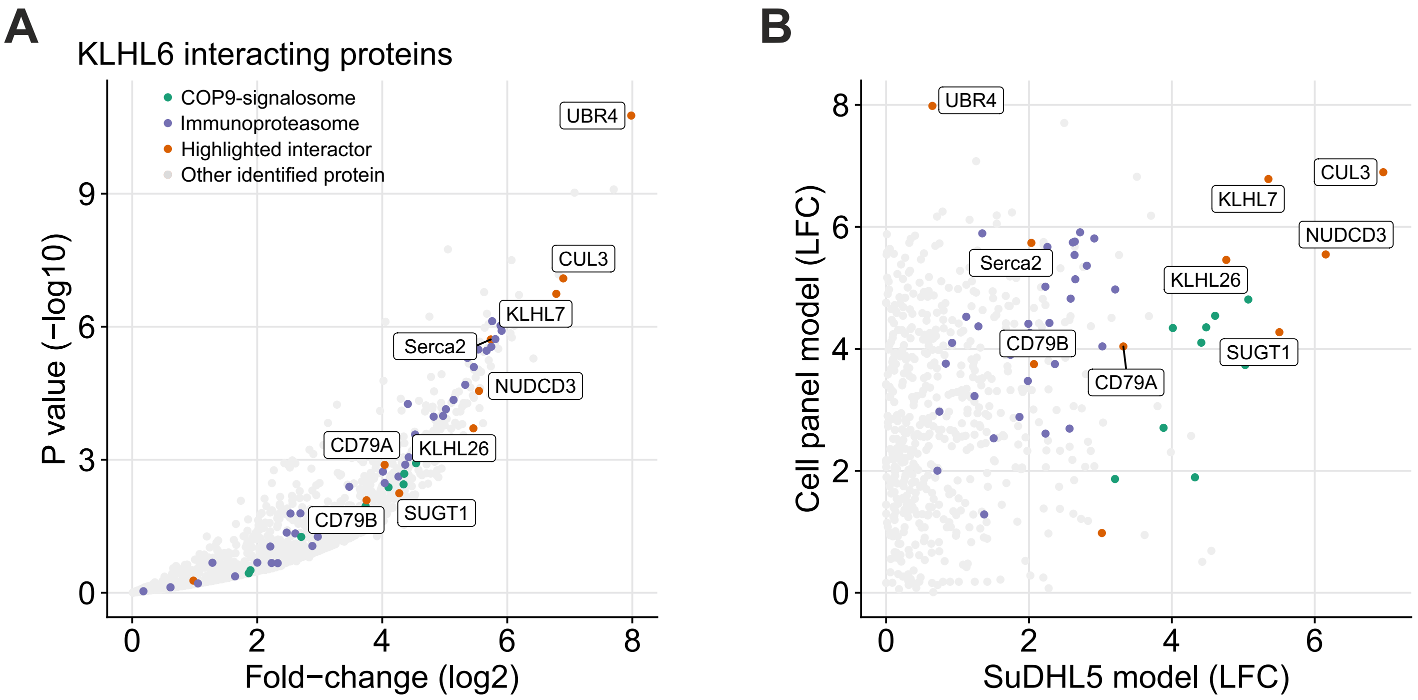
A
KLHL6 interacting proteins
COP9-signalosome
Immunoproteasome
Highlighted interactor
Other identified protein
UBR4
CUL3
KLHL7
Serca2
NUDCD3
CD79A
KLHL26
CD79B
SUGT1
P value (−log10)
Fold−change (log2)
B
UBR4
CUL3
KLHL7
NUDCD3
Serca2
KLHL26
CD79B
CD79A
SUGT1
Cell panel model (LFC)
SuDHL5 model (LFC)

Figure S4. Proximity-labeling interactome analysis of KLHL6.

(A) Representative fluorescent image of SuDHL5 cells stained for endogenous expression of KLHL6 (green), SEC16A (magenta) and DNA (DAPI). Individual channels for dashed area show on the right, acquired with 63x objective.

(B) Comparison of KLHL6 proximity-labeling interactomes between SuDHL5 and U2932 cell lines. X-axis, log2-transformed fold change (LFC) of spectral counts between EmGFP-BioID2 and KLHL6-BioID2 in U2932 cells (one biological replicate). Y-axis, LFC of normalized spectral counts between EmGFP-BioID2 and KLHL6-BioID2 in SuDHL5 cells (DESeq2 model, from Figure 4B x-axis).

(C) Western blot quantifications of BANK1 and LUZP1 in SuDHL5 with different KLHL6 modifications (KLHL6, KLHL6 overexpression; KO, knock-out; KD, knock-down). The quantified signal was normalized to β-actin and is shown in relation to the respective controls (EmGFP overexpression, CRISPR-Cas9 control guide RNA).

Figure S4.

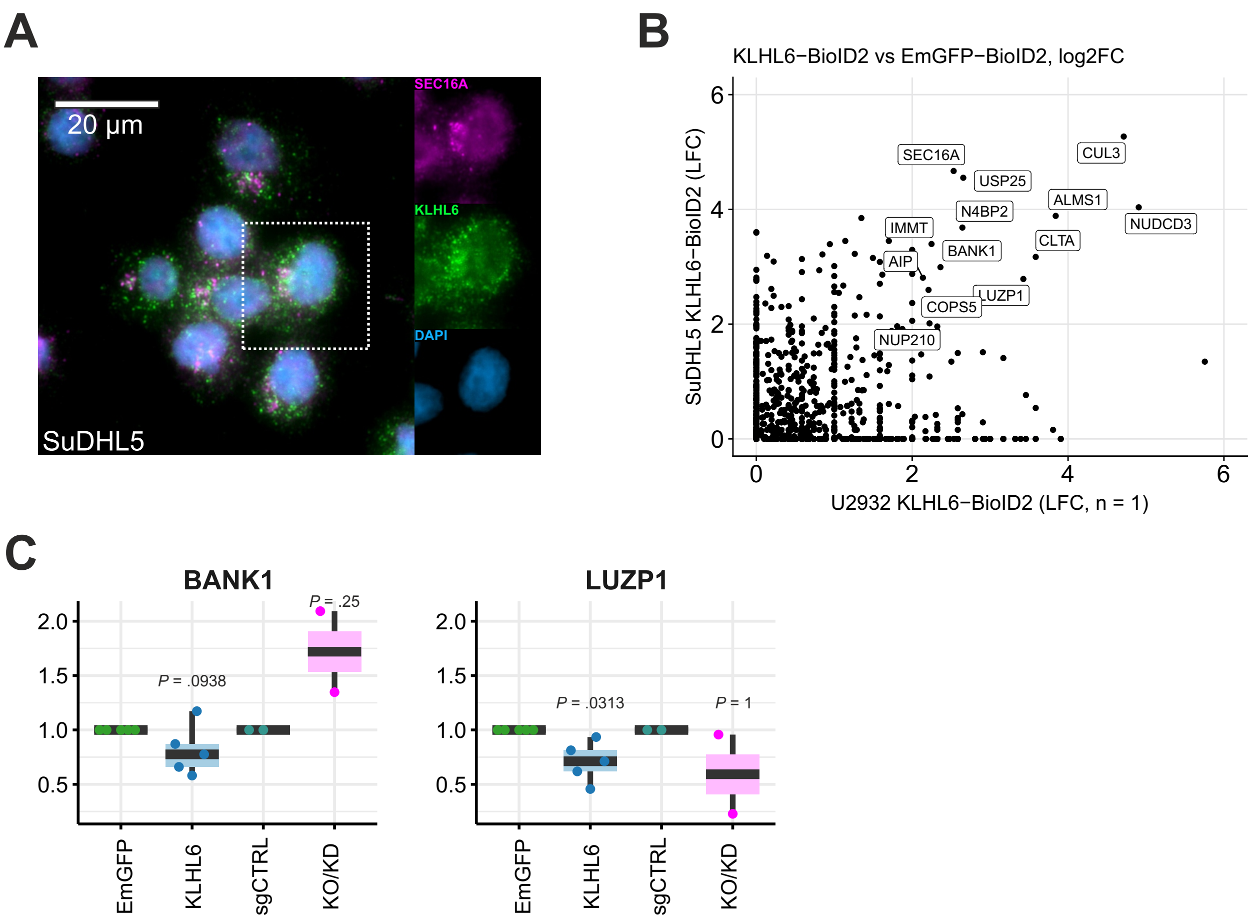

A
20 µm
SEC16A
KLHL6
DAPI
SuDHL5
B
KLHL6−BioID2 vs EmGFP−BioID2, log2FC
SuDHL5 KLHL6−BioID2 (LFC)
U2932 KLHL6−BioID2 (LFC, n = 1)
0
2
4
6
SEC16A
CUL3
USP25
N4BP2
ALMS1
IMMT
NUDCD3
CLTA
BANK1
AIP
LUZP1
COPS5
NUP210
C
BANK1
LUZP1
2.0
1.5
1.0
0.5
P = .0938
P = .25
P = .0313
P = 1
EmGFP
KLHL6
sgCTRL
KO/KD

Figure S5. KLHL6 interactome upon BCR stimulation and the impact of recurrent mutations on BCR levels.

(A) Ca^{2+} flux kinetics after stimulation with goat anti-human IgM F(ab')2 or unspecific goat IgG in SuDHL5 cells. X-axis, time in seconds. Y-axis, measured fluorescence signal of IgM F(ab')2 stimulated cells per signal of unspecific goat IgG treated cells at the same time point. Time points (vertical lines) indicating start of stimulation and peak fluorescent signal intensity after 60 seconds from stimulation.

(B) Western blot of cytosolic cell lysates from SuDHL5 cells stimulated or control treated for affinity-purification (AP) experiments upon BCR cross-linking. Four replicates of SuDHL5 cells treated with goat anti-human IgM F(ab')2 or unspecific goat IgG. Red bands indicate quality control of successful stimulation *in vitro* (phosphorylated SYK, p-SYK). Green bands, housekeeping loading control.

(C) Dot plot showing differences in KLHL6 AP interactome between BCR-stimulated and control treated SuDHL5 cells. X-axis is the log2 fold-change (LFC) in DESeq2 model between stimulation and control treatment with positive LFC signaling stronger interaction upon BCR engagement. Y-axis represents the LFC of KLHL6 interacting proteins against control purifications in DeSEQ2 model from Figure 4B.

(D) Box and dot plot of non-normalized spectral counts of pooled proteasome component proteins with different baits and conditions. Mann-Whitney U *P* values.

(E) Bar plot showing western blot quantifications (y-axis) of CD79A and CD79B from SuDHL5 cells overexpressing KLHL6, V5-tagged KLHL7 or KLHL26, or EmGFP (CTRL). Quantified signal was normalized to β-actin normalized to the EmGFP.

(F) Bar plot of western blot quantifications (y-axis) of CD79A and CD79B from Cas9 expressing SuDHL4 cells transduced with KLHL6 or KLHL7 targeting sgRNAs or control sgRNAs targeting chromosome 2 (Chr2) or MYC. Quantified signal was normalized to total protein load and is shown in relation to the Chr2 control.

(G) Western blot showing the impact of KLHL6 or KLHL7 silencing on CD79A and CD79B levels in single cell cloned Cas9-expressing SuDHL4 cells. Silencing of KLHL7 was confirmed with capillary sequencing. Quantified protein levels normalized to total protein load are shown in the heatmaps below the blot.

(H) Western blot showing the impact of wild-type or mutant KLHL6 constructs on CD79A, CD79B, and IgM levels in Ocily7 cells. Quantified protein levels normalized to total protein load are shown in the heatmaps below the blot.

(I) Bar plots showing western blot quantifications of CD79A, CD79B, and IgH (IgM/IgG) in SuDHL5, SuDHL4, U2932, and HBL1 cell lines transduced with different KLHL6 or control (EmGFP) constructs, Quantified signal was normalized to total protein load and is shown relative to EmGFP control.

Figure S5.

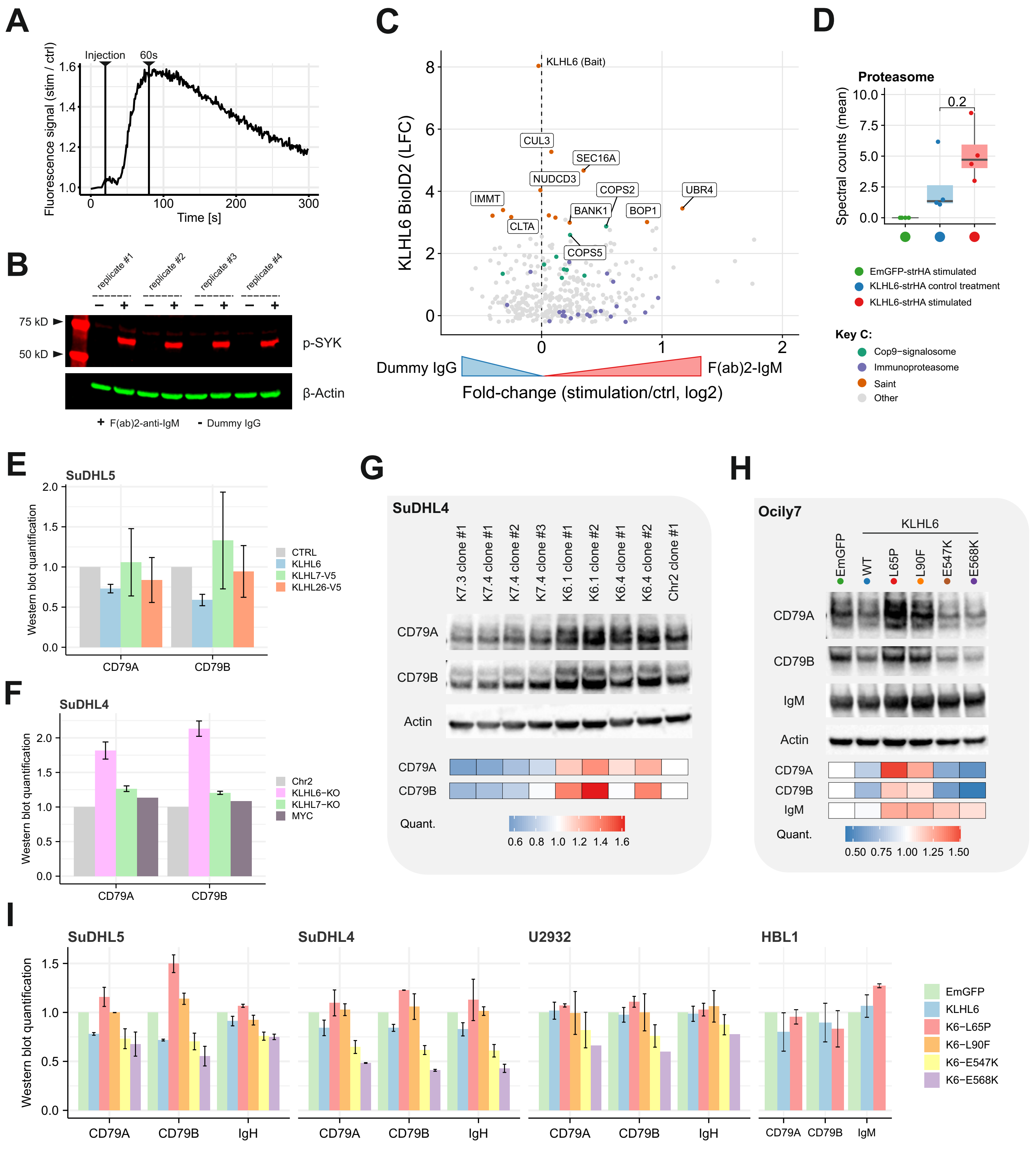
A
Injection
60s
Fluorescence signal (stim / ctrl)
Time [s]
B
replicate #1
replicate #2
replicate #3
replicate #4
75 kD
50 kD
p-SYK
β-Actin
+ F(ab)2-anti-IgM
- Dummy IgG
C
KLHL6 (Bait)
CUL3
SEC16A
NUDCD3
IMMT
COPS2
UBR4
BANK1
BOP1
CLTA
COPS5
KLHL6 BioID2 (LFC)
Dummy IgG
F(ab)2-IgM
Fold-change (stimulation/ctrl, log2)
D
Proteasome
Spectral counts (mean)
0.2
EmGFP-strHA stimulated
KLHL6-strHA control treatment
KLHL6-strHA stimulated
Key C:
Cop9−signalosome
Immunoproteasome
Saint
Other
E
SuDHL5
Western blot quantification
CTRL
KLHL6
KLHL7-V5
KLHL26-V5
CD79A
CD79B
F
SuDHL4
Western blot quantification
Chr2
KLHL6−KO
KLHL7−KO
MYC
CD79A
CD79B
G
SuDHL4
K7.3 clone #1
K7.4 clone #1
K7.4 clone #2
K7.4 clone #3
K6.1 clone #1
K6.1 clone #2
K6.4 clone #1
K6.4 clone #2
Chr2 clone #1
CD79A
CD79B
Actin
Quant.
H
Ocily7
KLHL6
EmGFP
WT
L65P
L90F
E547K
E568K
CD79A
CD79B
IgM
Actin
Quant.
I
SuDHL5
SuDHL4
U2932
HBL1
Western blot quantification
CD79A
CD79B
IgH
IgM
EmGFP
KLHL6
K6−L65P
K6−L90F
K6−E547K
K6−E568K

Figure S6. Impact of KLHL6 constructs on the surface levels expression of the BCR components. Flow cytometry analysis showing impacts of different KLHL6 constructs on surface levels of all BCR components in DLBCL cell lines without altering CD19 surface expression. Genetically unmanipulated 'parental' cell line is used as control. Values on x-axis represent archsinh ratio of MFI normalized to expression in parental cells. Histograms show data from one representative experiment, box and dot plots show combined data from four (SuDHL5) and five experiments (SuDHL4, Ocily7 and U2932). *P* values calculated with two-sided one-sample t-test.

Figure S6.

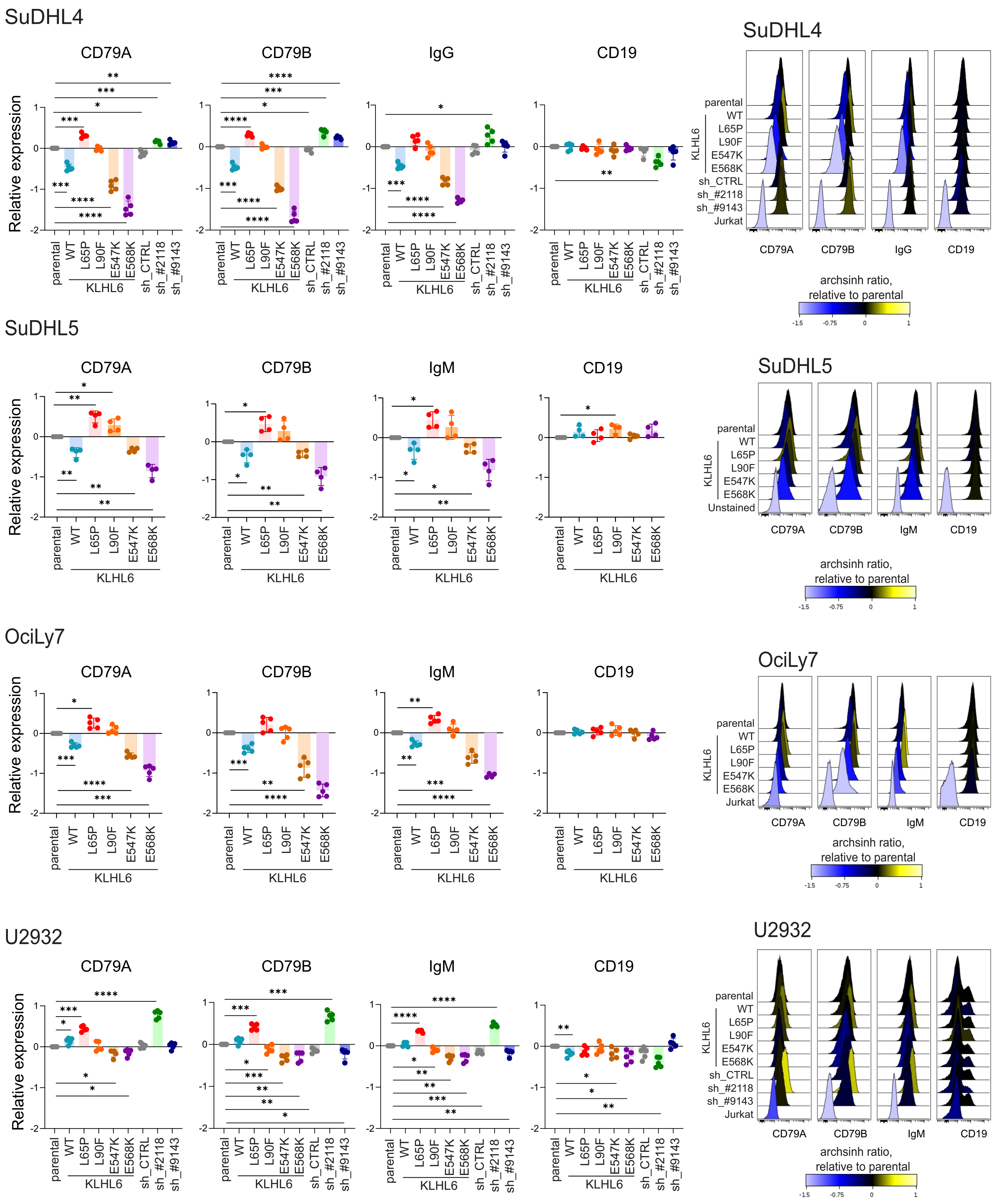
SuDHL4
CD79A
CD79B
IgG
CD19
Relative expression
parental
WT
L65P
L90F
E547K
E568K
sh_CTRL
sh_#2118
sh_#9143
KLHL6
Jurkat
archsinh ratio, relative to parental
SuDHL5
IgM
Unstained
OciLy7
U2932

Figure S7. Impact of KLHL6 constructs on the BCR signaling.

(A) Histograms of anti-IgG or anti-IgM-induced signaling in parental and genetically modified DLBCL cells overexpressing KLHL6 wild-type, mutant constructs, or shRNA knock-down of *KLHL6*, measured by phospho-flow. Phosphorylated levels of SYK, Src family kinases (SFK), BTK, PLCγ, BLNK, ERK, AKT, STAT5 and p38 are shown as arcsinh ratio of MFI normalized to expression in unstimulated cells. Histograms showing average from four (SuDHL5) and five experiments (SuDHL4, OciLy and U2932), and the anti-IgG $F(ab')_2$ or anti-IgM $F(ab')_2$ concentrations used for activation are indicated. *P* values were calculated with one-way ANOVA followed by Dunnett's multiple comparisons test against the unstimulated control.

(B) Line graph showing cell viability (y-axis) of SuDHL5 or U2932 cells transduced with distinct KLHL6 constructs or EmGFP control construct. *KLHL6* knock-out (KO) included for SuDHL5. Measured luminescence signal per measurement was normalized to the first measurement at 24-hour time point.

(C) Line and dot graph showing viability (y-axis) of SuDHL5 cells with KLHL6 overexpression or KLHL6 knock-out or their respective controls (EmGFP or sgCTRL) 48 hours after B-cell receptor stimulation with anti-IgM different concentrations (x-axis). Measured signal was normalized to control treatment with unspecific goat IgG.

Figure S7.

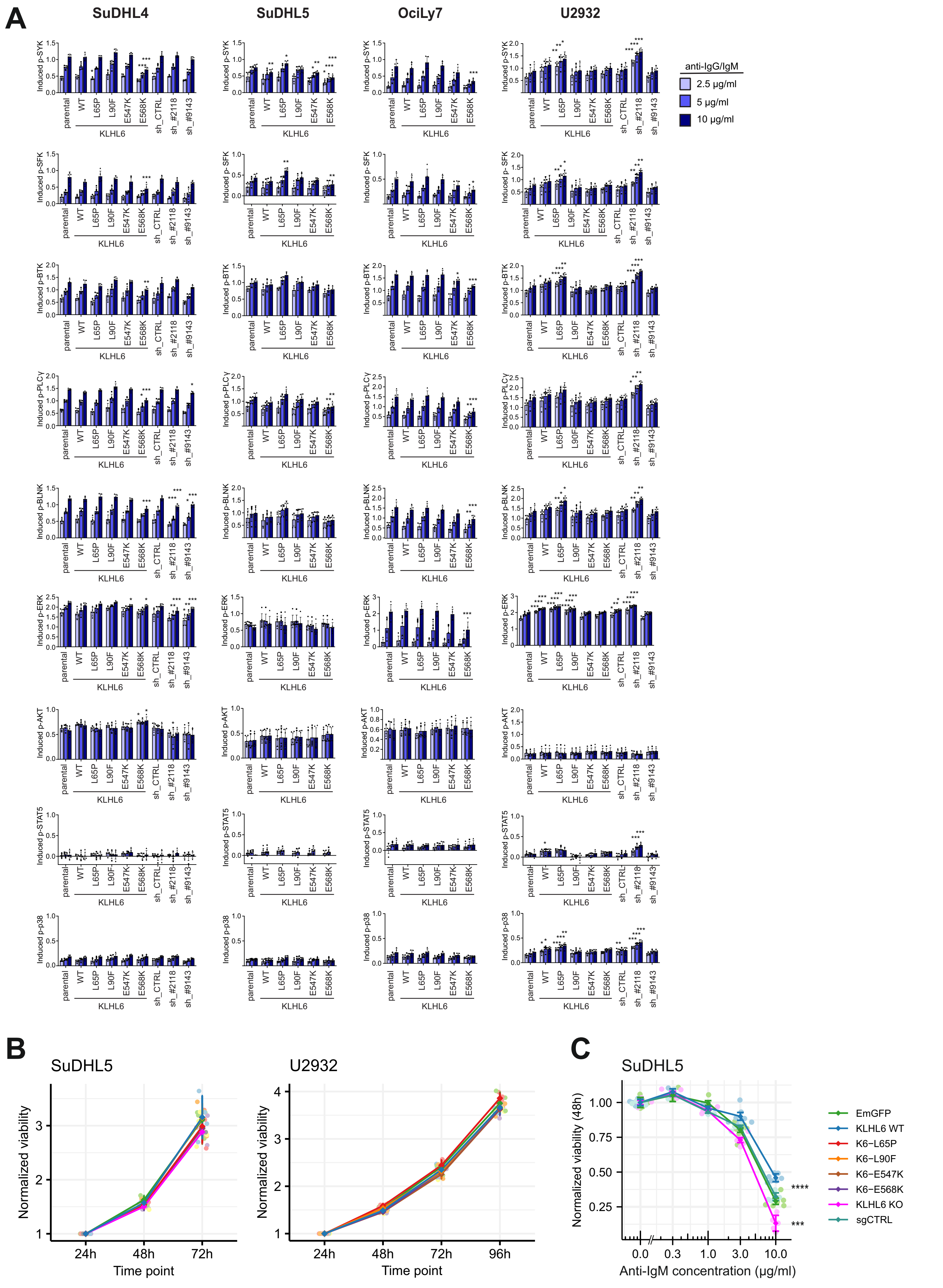
A
SuDHL4
SuDHL5
OciLy7
U2932
anti-IgG/IgM
2.5 µg/ml
5 µg/ml
10 µg/ml
Induced p-SYK
Induced p-SFK
Induced p-BTK
Induced p-PLCγ
Induced p-BLNK
Induced p-ERK
Induced p-AKT
Induced p-STAT5
Induced p-p38
parental
WT
L65P
L90F
E547K
E568K
sh_CTRL
sh_#2118
sh_#9143
KLHL6
B
SuDHL5
U2932
Normalized viability
24h
48h
72h
96h
Time point
C
SuDHL5
Normalized viability (48h)
Anti-IgM concentration (µg/ml)
EmGFP
KLHL6 WT
K6−L65P
K6−L90F
K6−E547K
K6−E568K
KLHL6 KO
sgCTRL
****
***

Figure S8. Immunofluorescent analysis and quantification of CD79B staining intensity.

(A) Representative immunofluorescent (IF) microscopy image of a germinal center (GC) in reactive human tonsil showing highest CD79B intensity (magenta) in the mantle zone and strongest KLHL6 positivity (green) restricted to the germinal center. Scale bar 100 micrometers.

(B) Exemplar analysis of a tissue micro-array (TMA) scan with CD79B quantification of six different tumors. Mean fluorescent intensity of CD79B stain (Alexa Fluor 647, magenta) quantified for each representative TMA punch for the dashed region of interest (ROI). Final CD79B quantification was the mean from all representative punches. Bar plot on the right shows quantified CD79B intensity (y-axis), according to tumors (x-axis) and individual dots represent each measurement from the ROIs. White pseudocolor, DAPI.

(C) Box plots of quantified CD79B intensity (y-axis) according to different TMA slides (x-axis) scanned of the discovery cohort. Green dots indicate mean CD79B intensities per tumor sample (1-3 punches analyzed) and brown dots show background quantification of liver tissue punches (three on each slide slide). Numbers in parenthesis represent the number of tumors analyzed from each TMA slide.

(D) Dot and line graph of CD79B intensity (y-axis) and corresponding *CD79B* gene expression level (log2 FPKM, RNA sequencing data). Spearman correlation.

(E) Dot and line graph of CD79B intensity (y-axis) and corresponding *CD79B* gene expression level (log2 FPKM, RNA sequencing data) according to $KLHL6^{GC+}$ phenotype (color). Spearman correlation.

(F) Dot and box plots of CD79B intensity (y-axis) according to molecular subtype (x-axis). Mann-Whitney U test *P* value.

(G) Images of capillary (Sanger) sequencing histograms for select patients showing *KLHL6* mutations encoding a mutation in the BTB domain. These tumors were analyzed for CD79B and KLHL6 protein expression in Figure 6I. Mutations underlined.

(H) Interactive genomics viewer screenshot of a sequencing alignments from diagnostic tumor tissue of a patient with KLHL6 E547K mutation analyzed with IF microscopy in Figure 6I. The lane above is the tumor, below the matched whole blood of the patient.

Figure S8.

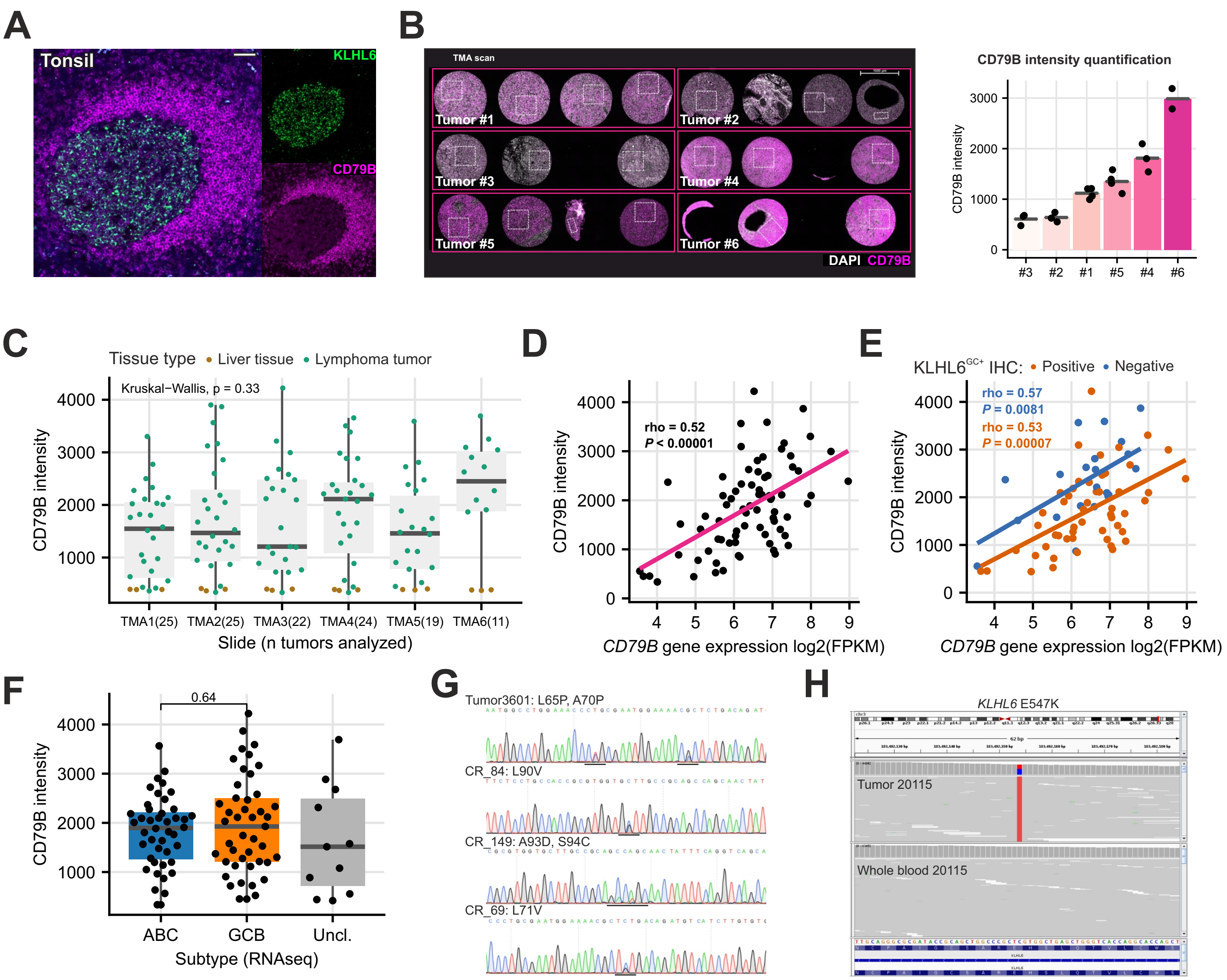

A
Tonsil
KLHL6
CD79B
B
TMA scan
Tumor #1
Tumor #2
Tumor #3
Tumor #4
Tumor #5
Tumor #6
DAPI CD79B
CD79B intensity quantification
CD79B intensity
#3 #2 #1 #5 #4 #6
C
Tissue type • Liver tissue • Lymphoma tumor
Kruskal−Wallis, p = 0.33
CD79B intensity
TMA1(25) TMA2(25) TMA3(22) TMA4(24) TMA5(19) TMA6(11)
Slide (n tumors analyzed)
D
rho = 0.52
P < 0.00001
CD79B intensity
CD79B gene expression log2(FPKM)
E
KLHL6GC+ IHC: • Positive • Negative
rho = 0.57
P = 0.0081
rho = 0.53
P = 0.00007
CD79B intensity
CD79B gene expression log2(FPKM)
F
0.64
CD79B intensity
ABC GCB Uncl.
Subtype (RNAseq)
G
Tumor3601: L65P, A70P
CR_84: L90V
CR_149: A93D, S94C
CR_69: L71V
H
KLHL6 E547K
Tumor 20115
Whole blood 20115

Figure S9. BANK1 expression in DLBCL.

(A) Western blot showing BANK1 and CD79A levels in DLBCL cell lines. Arrowheads indicate different BANK1 isoforms.

(B) UMAP visualization of 624 DLBCL transcriptomes. The color of the samples is according to BANK1 expression (log2-transformed FPKM).

(C–E) BANK1 transcript expression according to C) molecular subtypes D) *MYD88* mutation status and E) dominant B-cell states of tumors. Comparisons and *P* values according to Mann-Whitney U test (C and D) and Kruskal-Wallis test (E).

Figure S9.

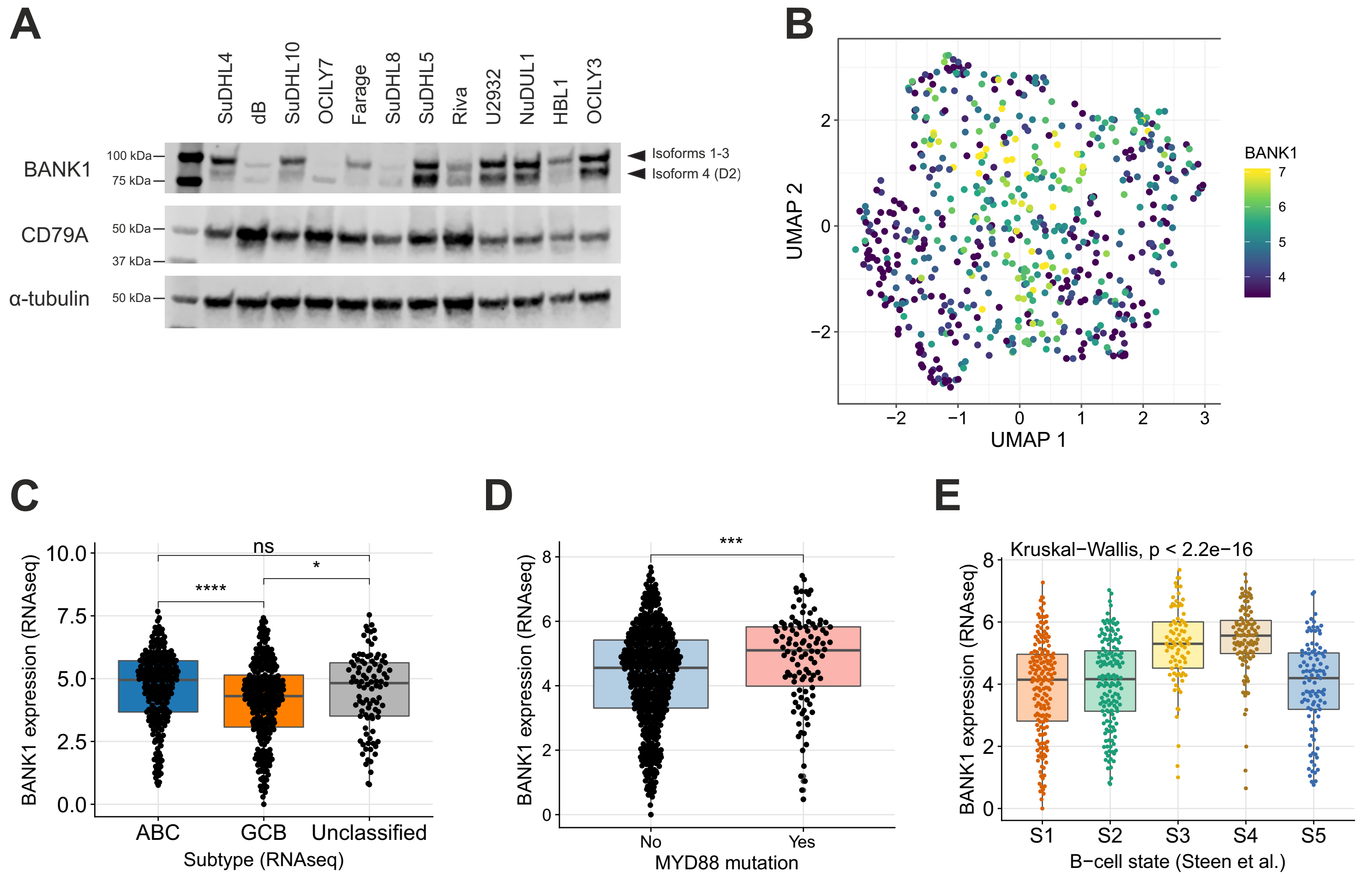
A
SuDHL4
dB
SuDHL10
OCILY7
Farage
SuDHL8
SuDHL5
Riva
U2932
NuDUL1
HBL1
OCILY3
BANK1
100 kDa
75 kDa
Isoforms 1-3
Isoform 4 (D2)
CD79A
50 kDa
37 kDa
α-tubulin
50 kDa
B
UMAP 2
UMAP 1
BANK1
7
6
5
4
C
BANK1 expression (RNAseq)
ns
*
****
ABC
GCB
Unclassified
Subtype (RNAseq)
D
BANK1 expression (RNAseq)
***
No
Yes
MYD88 mutation
E
Kruskal−Wallis, p < 2.2e−16
BANK1 expression (RNAseq)
S1
S2
S3
S4
S5
B−cell state (Steen et al.)
