# Supplementary material for: Disruption of KLHL6 Fuels Oncogenic Antigen Receptor Signaling in B-Cell Lymphoma
Source: Blood Cancer Discov. 2024 Apr 17;5(5):331–52. doi: 10.1158/2643-3230.BCD-23-0182 (PMC11369598; doi:10.1158/2643-3230.BCD-23-0182)
Supplement: Unprocessed Western blots — for Figures 1E, 3I, 4E, 4F, 5A, 5S5, 5E, 5F, 5G, 5H, 7D, 7K, S5G, S5H, and S9A [file bcd-23-0182_unprocessed_western_blots_suppsf1e-sf9a.pdf]

Supplementary file: Unprocessed western blots

’Disruption of KLHL6 Fuels Oncogenic Antigen Receptor Signaling in B-cell Lymphoma’ Meriranta et al. 2024

Figure 1E

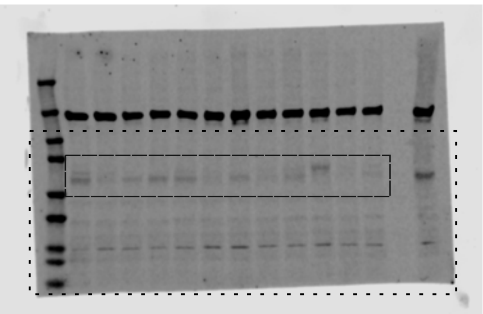

KLHL6

Imaging system: Odyssey Fc Imaging System
Channel: 700
Software: Image Studio Lite

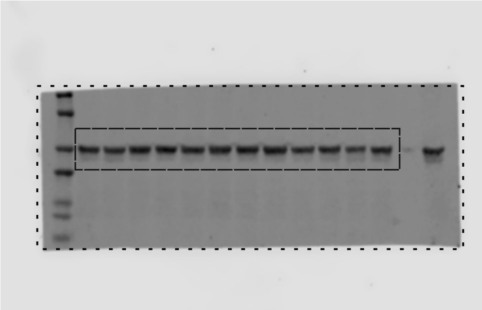

α-Tubulin

Imaging system: Odyssey Fc Imaging System
Channel: 700
Software: Image Studio Lite

Figure 3I

Lysates

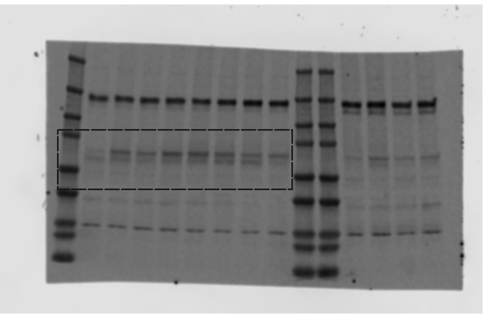

KLHL6

Imaging system: Odyssey Fc Imaging System
Channel: 700
Software: Image Studio Lite

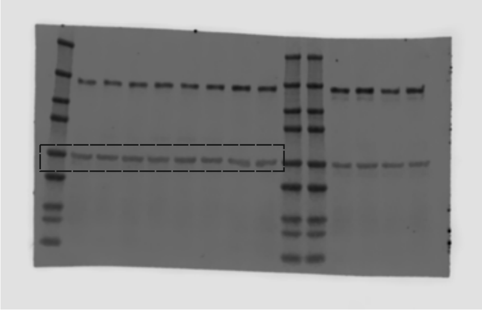

α-Tubulin

Imaging system: Odyssey Fc Imaging System
Channel: 700
Software: Image Studio Lite

Eluates

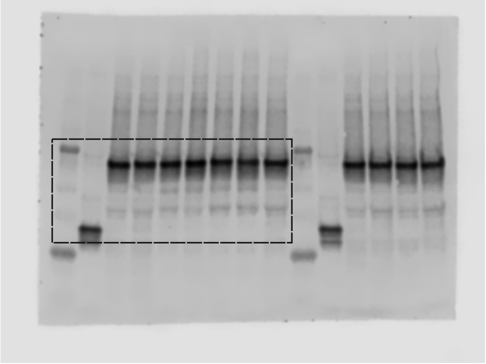

HA-tag

Imaging system: Odyssey Fc Imaging System
Channel: 800
Software: Image Studio Lite

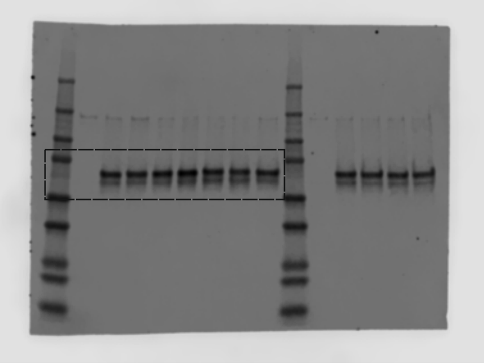

KLHL6

Imaging system: Odyssey Fc Imaging System
Channel: 700
Software: Image Studio Lite

Figure 4E

SuDHL5

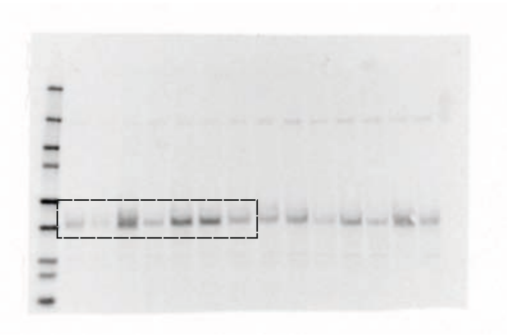

CD79B

Imaging system: Azure 500 Imaging System
Channel: 680
Software: AzureSpot Pro

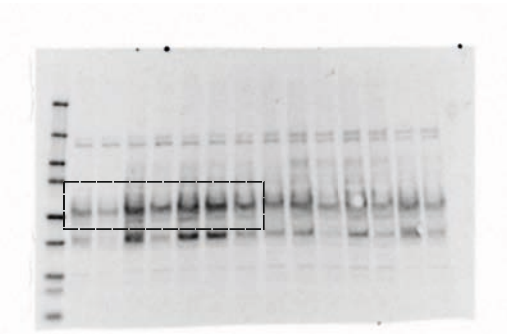

CD79A

Imaging system: Azure 500 Imaging System
Channel: 680
Software: AzureSpot Pro

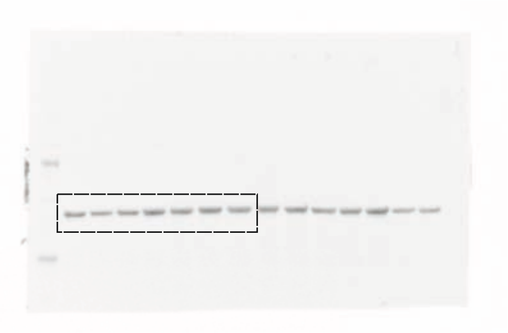

β-actin

Imaging system: Azure 500 Imaging System
Channel: 800
Software: AzureSpot Pro

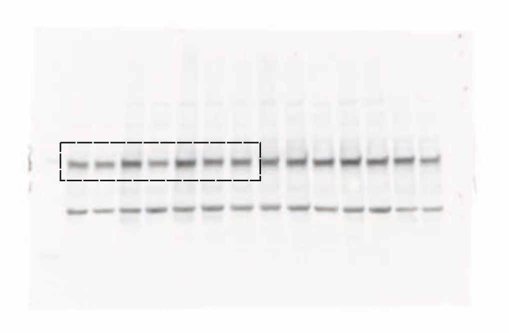

IgM

Imaging system: Azure 500 Imaging System
Channel: 800
Software: AzureSpot Pro

SuDHL4

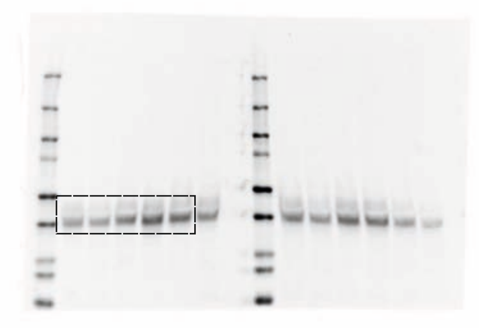

CD79B

Imaging system: Azure 500 Imaging System
Channel: 680
Software: AzureSpot Pro

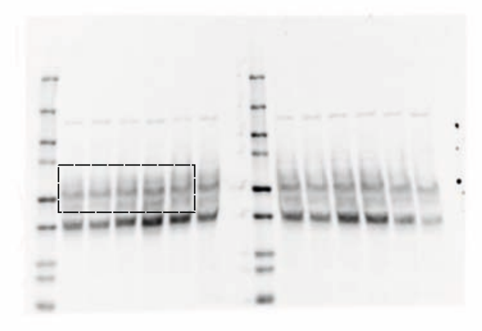

CD79A

Imaging system: Azure 500 Imaging System
Channel: 680
Software: AzureSpot Pro

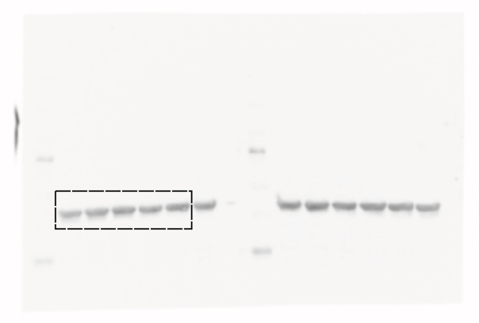

β-actin

Imaging system: Azure 500 Imaging System
Channel: 800
Software: AzureSpot Pro

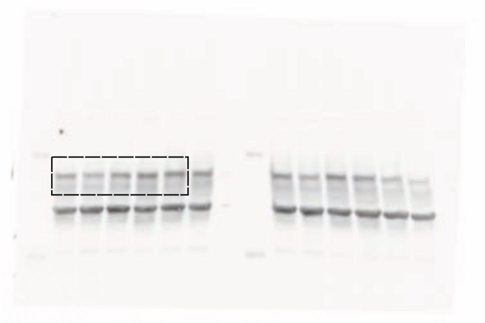

IgG

Imaging system: Azure 500 Imaging System
Channel: 800
Software: AzureSpot Pro

Figure 4E

U2932

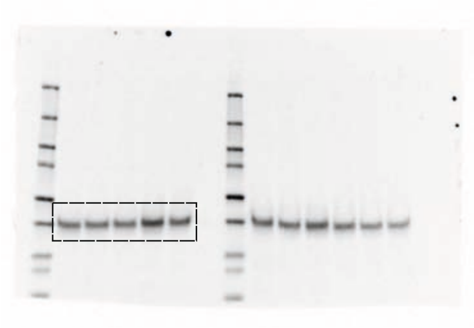

CD79B

Imaging system: Azure 500 Imaging System
Channel: 680
Software: AzureSpot Pro

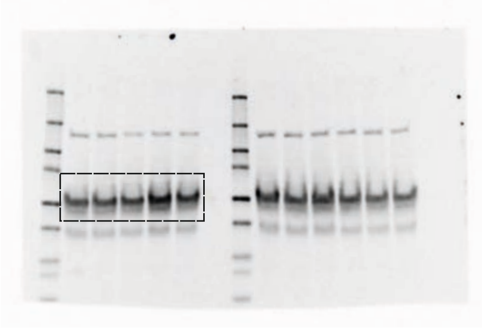

CD79A

Imaging system: Azure 500 Imaging System
Channel: 680
Software: AzureSpot Pro

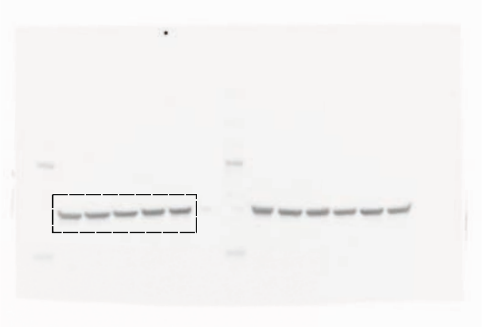

β-actin

Imaging system: Azure 500 Imaging System
Channel: 800
Software: AzureSpot Pro

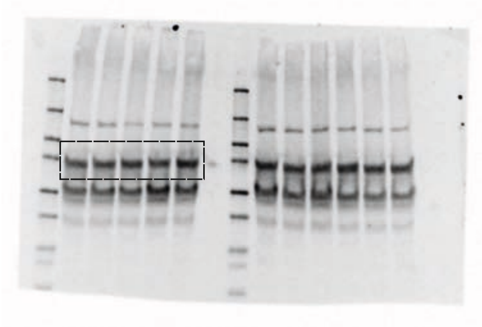

IgM

Imaging system: Azure 500 Imaging System
Channel: 800
Software: AzureSpot Pro

HBL1

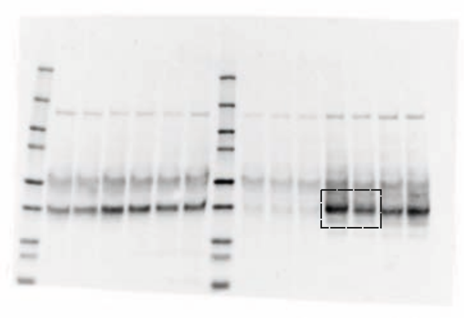

CD79B

Imaging system: Azure 500 Imaging System
Channel: 680
Software: AzureSpot Pro

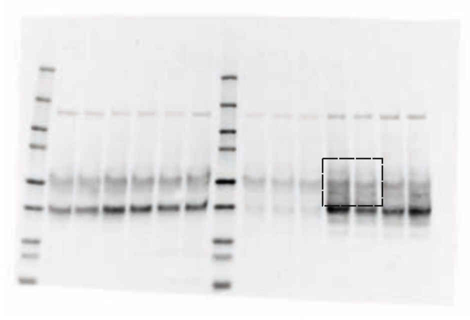

CD79A

Imaging system: Azure 500 Imaging System
Channel: 680
Software: AzureSpot Pro

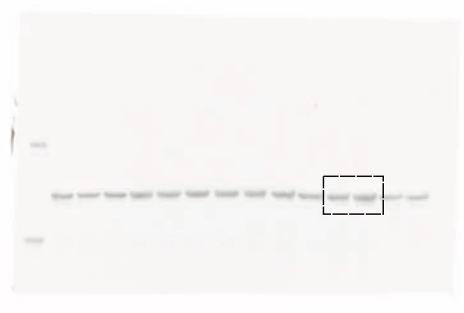

β-actin

Imaging system: Azure 500 Imaging System
Channel: 800
Software: AzureSpot Pro

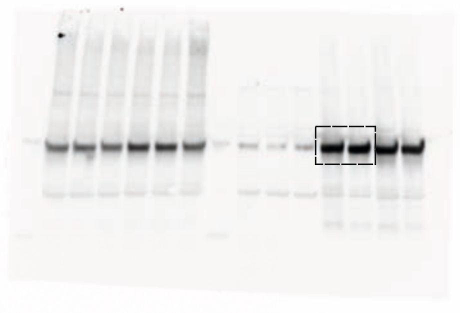

IgM

Imaging system: Azure 500 Imaging System
Channel: 800
Software: AzureSpot Pro

Figure 4F

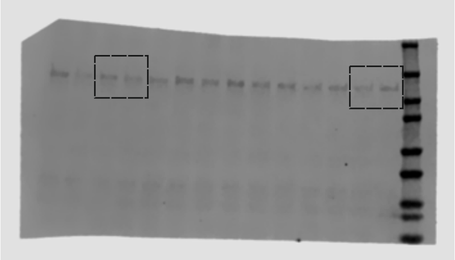

LuZP1

Imaging system: Odyssey Fc Imaging System
Channel: 700
Software: Image Studio Lite

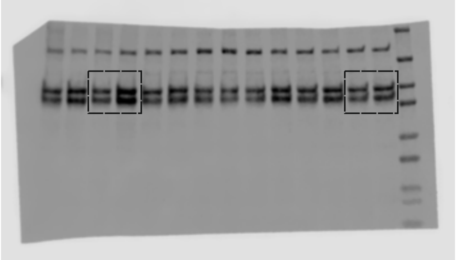

BANK1

Imaging system: Odyssey Fc Imaging System
Channel: 700
Software: Image Studio Lite

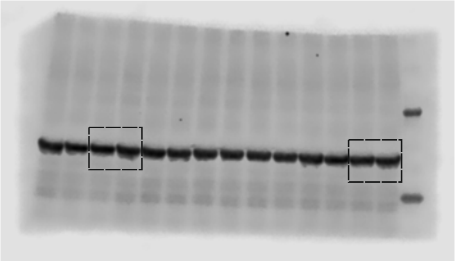

β-actin

Imaging system: Odyssey Fc Imaging System
Channel: 800
Software: Image Studio Lite

Figure 5A and S5B

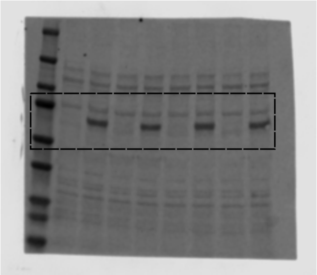

p-SYK

Imaging system: Odyssey Fc Imaging System
Channel: 700
Software: Image Studio Lite

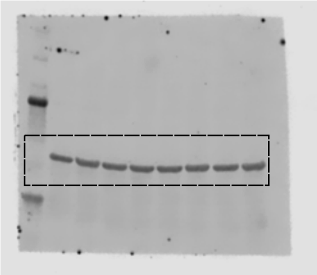

β-actin

Imaging system: Odyssey Fc Imaging System
Channel: 800
Software: Image Studio Lite

Figure 5E

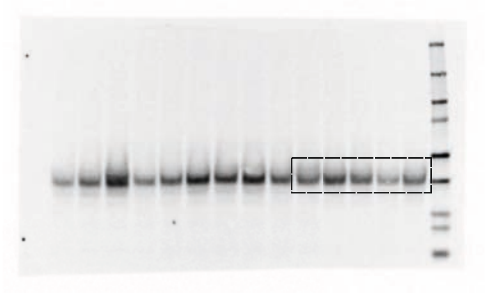

CD79B

Imaging system: Azure 500 Imaging System
Channel: 680
Software: AzureSpot Pro

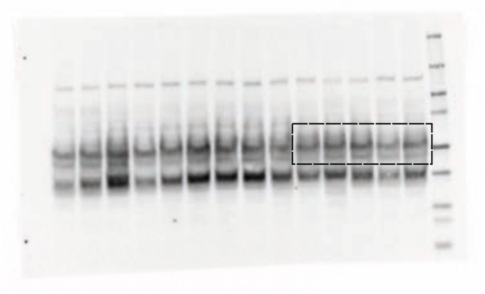

CD79A

Imaging system: Azure 500 Imaging System
Channel: 680
Software: AzureSpot Pro

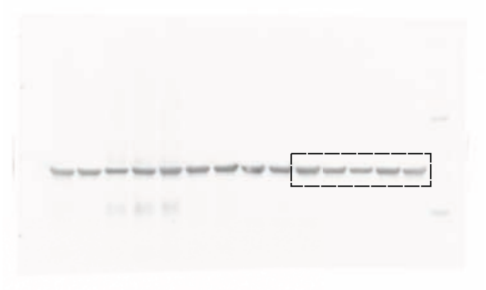

β-actin

Imaging system: Azure 500 Imaging System
Channel: 800
Software: AzureSpot Pro

Figure 5F

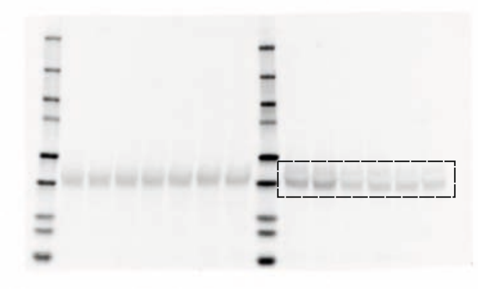

CD79B

Imaging system: Azure 500 Imaging System
Channel: 680
Software: AzureSpot Pro

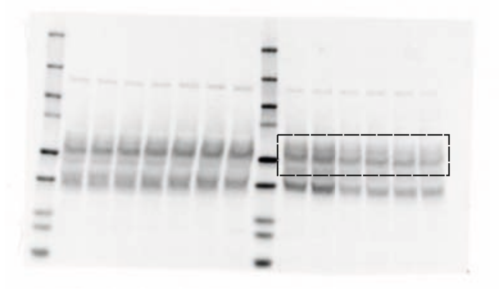

CD79A

Imaging system: Azure 500 Imaging System
Channel: 680
Software: AzureSpot Pro

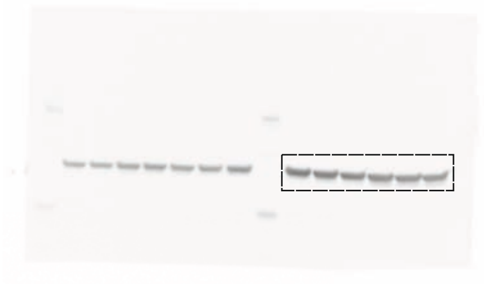

β-actin

Imaging system: Azure 500 Imaging System
Channel: 800
Software: AzureSpot Pro

Figure 5G

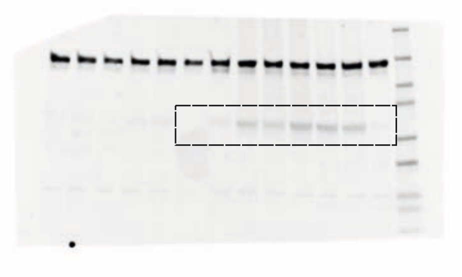

KLHL6

Imaging system: Azure 500 Imaging System
Channel: 680
Software: AzureSpot Pro

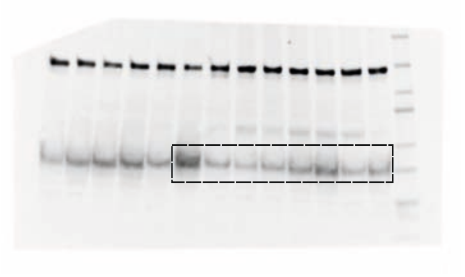

CD79B

Imaging system: Azure 500 Imaging System
Channel: 680
Software: AzureSpot Pro

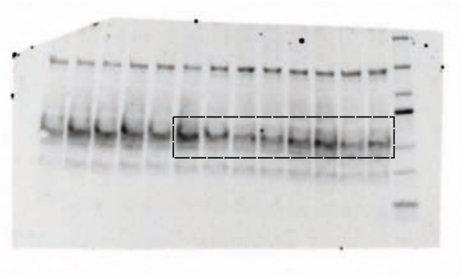

CD79A

Imaging system: Azure 500 Imaging System
Channel: 680
Software: AzureSpot Pro

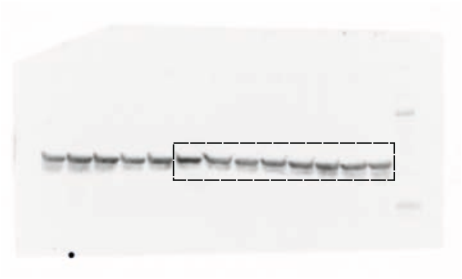

β-actin

Imaging system: Azure 500 Imaging System
Channel: 800
Software: AzureSpot Pro

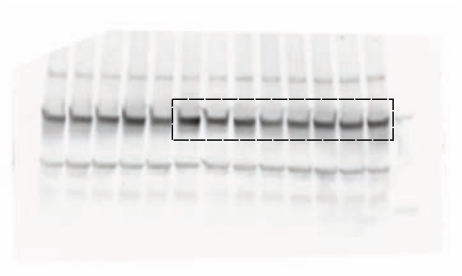

IgM

Imaging system: Azure 500 Imaging System
Channel: 800
Software: AzureSpot Pro

Figure 5H

SuDHL4

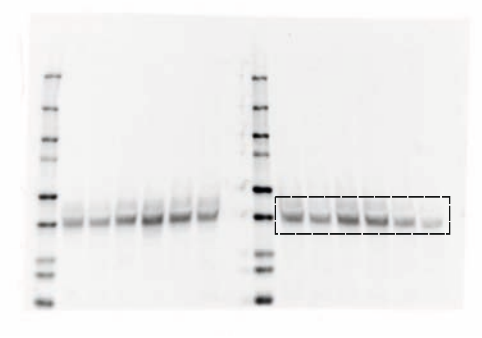

CD79B

Imaging system: Azure 500 Imaging System
Channel: 680
Software: AzureSpot Pro

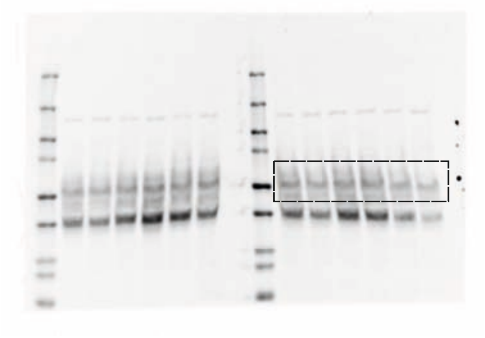

CD79A

Imaging system: Azure 500 Imaging System
Channel: 680
Software: AzureSpot Pro

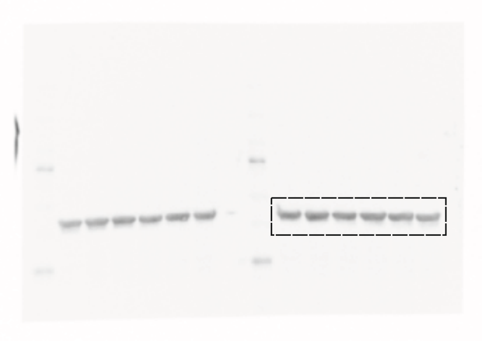

β-actin

Imaging system: Azure 500 Imaging System
Channel: 800
Software: AzureSpot Pro

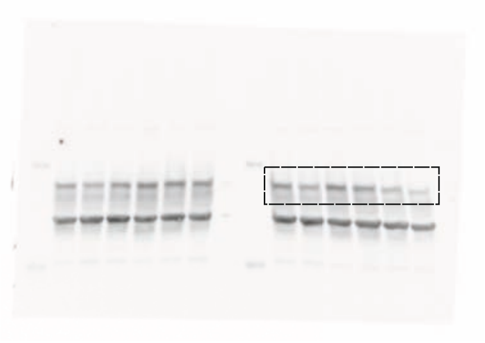

IgG

Imaging system: Azure 500 Imaging System
Channel: 800
Software: AzureSpot Pro

U2932

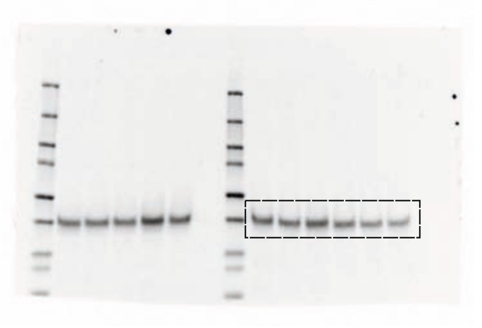

CD79B

Imaging system: Azure 500 Imaging System
Channel: 680
Software: AzureSpot Pro

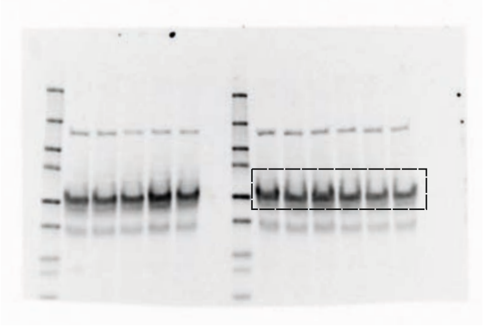

CD79A

Imaging system: Azure 500 Imaging System
Channel: 680
Software: AzureSpot Pro

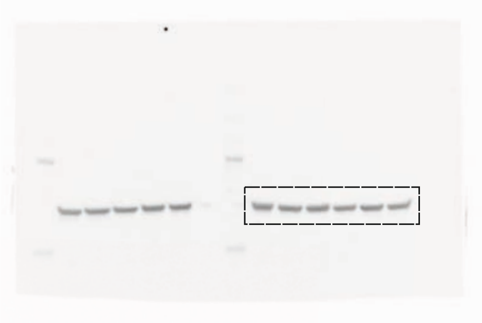

β-actin

Imaging system: Azure 500 Imaging System
Channel: 800
Software: AzureSpot Pro

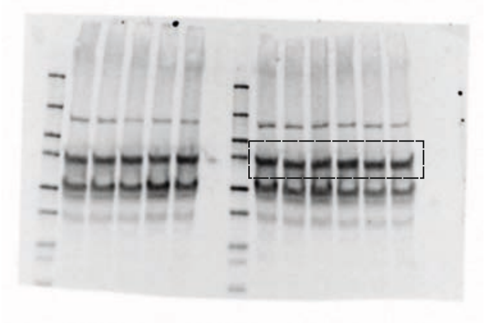

IgM

Imaging system: Azure 500 Imaging System
Channel: 680
Software: AzureSpot Pro

Figure 5H

HBL1

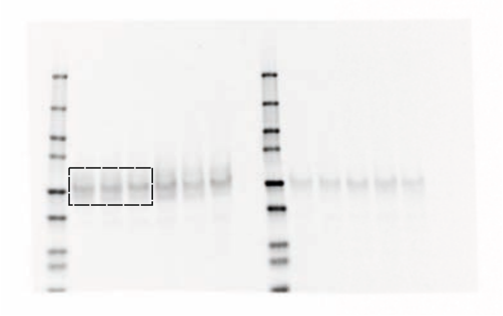

CD79A

Imaging system: Azure 500 Imaging System
Channel: 680
Software: AzureSpot Pro

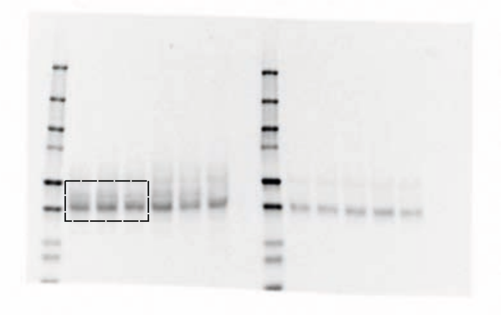

CD79B

Imaging system: Azure 500 Imaging System
Channel: 680
Software: AzureSpot Pro

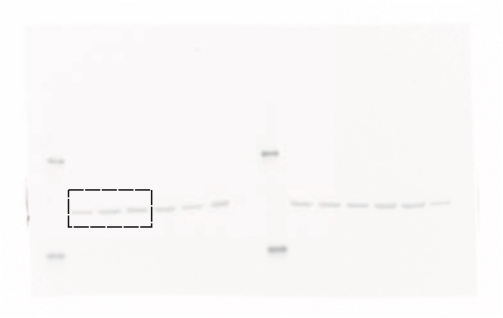

β-actin

Imaging system: Azure 500 Imaging System
Channel: 800
Software: AzureSpot Pro

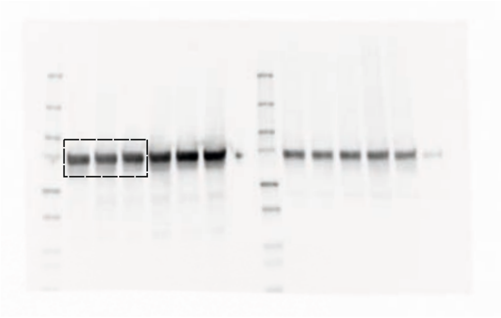

IgM

Imaging system: Azure 500 Imaging System
Channel: 680
Software: AzureSpot Pro

Figure 7D

SuDHL4

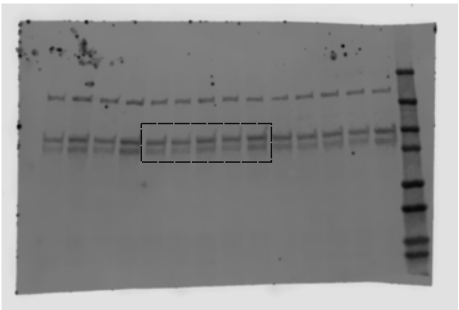

BANK1

Imaging system: Odyssey Fc Imaging System
Channel: 700
Software: Image Studio Lite

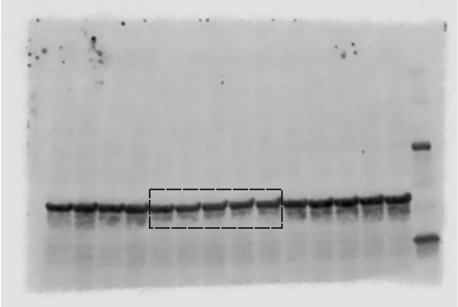

β-actin

Imaging system: Odyssey Fc Imaging System
Channel: 800
Software: Image Studio Lite

Figure 7D

SuDHL5

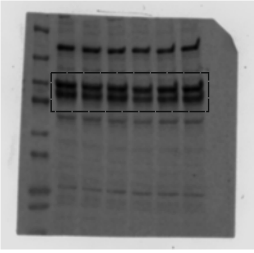

BANK1

Imaging system: Odyssey Fc Imaging System
Channel: 700
Software: Image Studio Lite

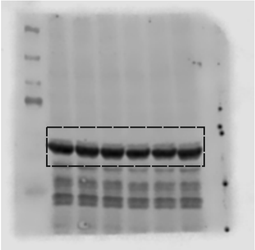

β-actin

Imaging system: Odyssey Fc Imaging System
Channel: 800
Software: Image Studio Lite

U2932

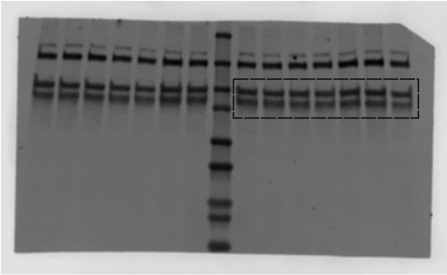

BANK1

Imaging system: Odyssey Fc Imaging System
Channel: 700
Software: Image Studio Lite

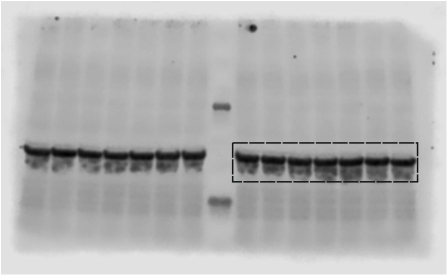

β-actin

Imaging system: Odyssey Fc Imaging System
Channel: 800
Software: Image Studio Lite

Figure 7K

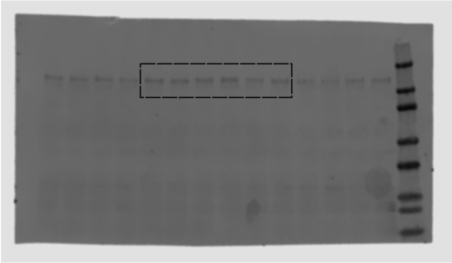

LuZP1

Imaging system: Odyssey Fc Imaging System
Channel: 700
Software: Image Studio Lite

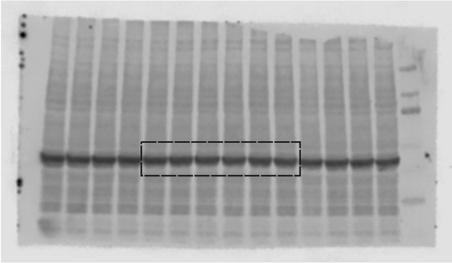

β-actin

Imaging system: Odyssey Fc Imaging System
Channel: 800
Software: Image Studio Lite

Figure S5G

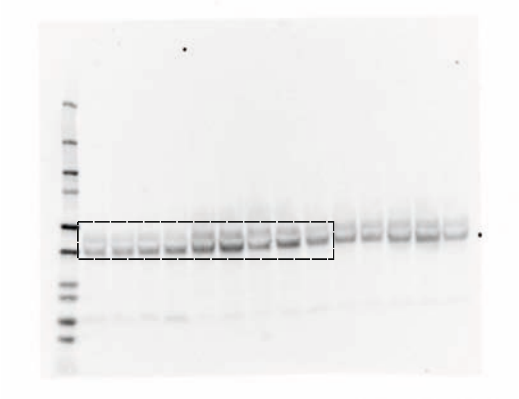

CD79B

Imaging system: Azure 500 Imaging System
Channel: 680
Software: AzureSpot Pro

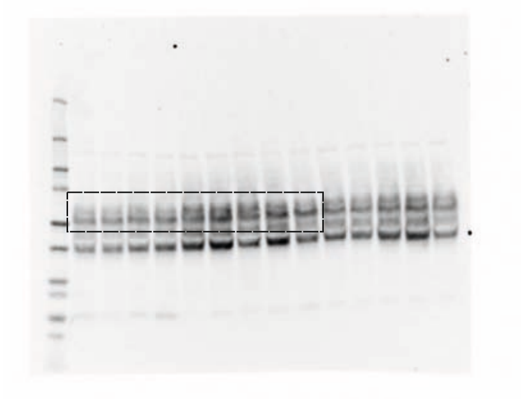

CD79A

Imaging system: Azure 500 Imaging System
Channel: 680
Software: AzureSpot Pro

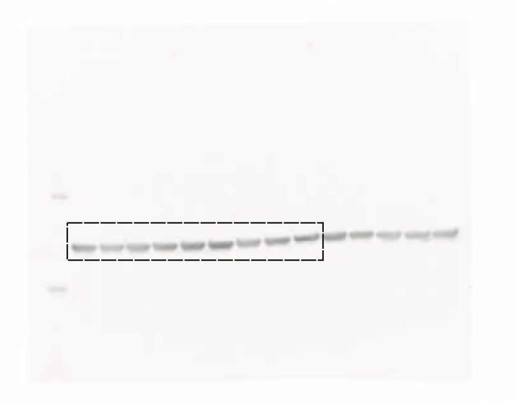

β-actin

Imaging system: Azure 500 Imaging System
Channel: 800
Software: AzureSpot Pro

Figure S5H

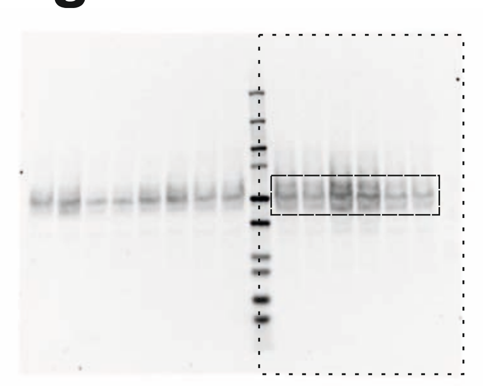

CD79A

Imaging system: Azure 500 Imaging System
Channel: 680
Software: AzureSpot Pro

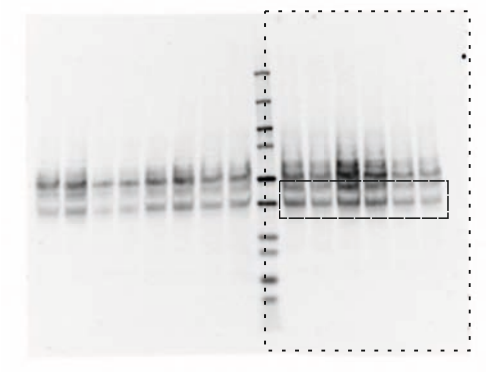

CD79B

Imaging system: Azure 500 Imaging System
Channel: 680
Software: AzureSpot Pro

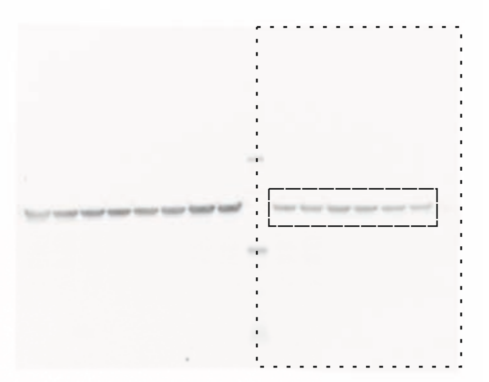

β-actin

Imaging system: Azure 500 Imaging System
Channel: 800
Software: AzureSpot Pro

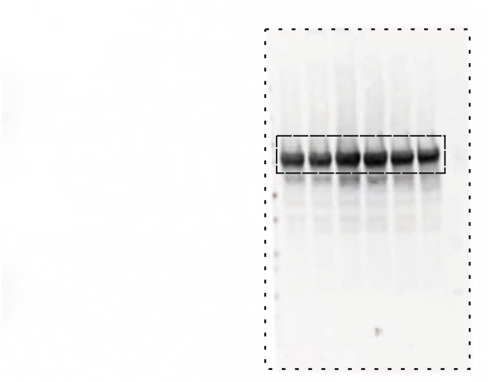

IgM

Imaging system: Azure 500 Imaging System
Channel: 800
Software: AzureSpot Pro

Figure S9A

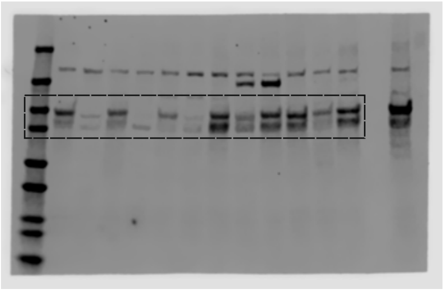

BANK1

Imaging system: Odyssey Fc Imaging System
Channel: 700
Software: Image Studio Lite

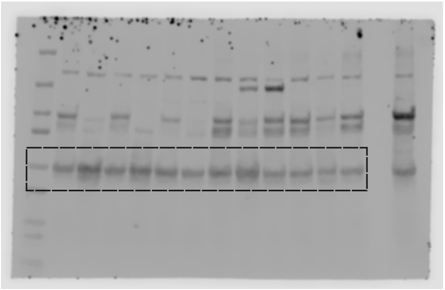

CD79A

Imaging system: Odyssey Fc Imaging System
Channel: 700
Software: Image Studio Lite

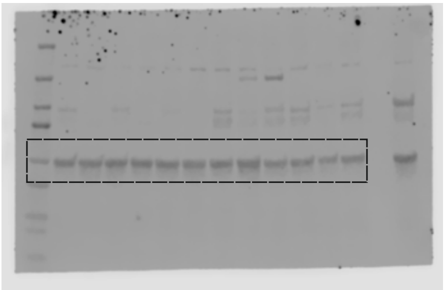

α-Tubulin

Imaging system: Odyssey Fc Imaging System
Channel: 700
Software: Image Studio Lite
